# Machine and cognitive intelligence for human health: systematic review

**DOI:** 10.1186/s40708-022-00153-9

**Published:** 2022-02-12

**Authors:** Xieling Chen, Gary Cheng, Fu Lee Wang, Xiaohui Tao, Haoran Xie, Lingling Xu

**Affiliations:** 1 Department of Mathematics and Information Technology, The Education University of Hong Kong, Hong Kong SAR, China; 2 School of Science and Technology, Hong Kong Metropolitan University, Hong Kong SAR, China; 3 School of Sciences, University of Southern Queensland, Toowoomba, Australia; 4 Department of Computing and Decision Sciences, Lingnan University, Hong Kong SAR, China

**Keywords:** Machine intelligence, Cognitive intelligence, Human health, Artificial intelligence, Systematic review

## Abstract

Brain informatics is a novel interdisciplinary area that focuses on scientifically studying the mechanisms of human brain information processing by integrating experimental cognitive neuroscience with advanced Web intelligence-centered information technologies. Web intelligence, which aims to understand the computational, cognitive, physical, and social foundations of the future Web, has attracted increasing attention to facilitate the study of brain informatics to promote human health. A large number of articles created in the recent few years are proof of the investment in Web intelligence-assisted human health. This study systematically reviews academic studies regarding article trends, top journals, subjects, countries/regions, and institutions, study design, artificial intelligence technologies, clinical tasks, and performance evaluation. Results indicate that literature is especially welcomed in subjects such as *medical informatics* and *health care sciences and service*. There are several promising topics, for example, random forests, support vector machines, and conventional neural networks for disease detection and diagnosis, semantic Web, ontology mining, and topic modeling for clinical or biomedical text mining, artificial neural networks and logistic regression for prediction, and convolutional neural networks and support vector machines for monitoring and classification. Additionally, future research should focus on algorithm innovations, additional information use, functionality improvement, model and system generalization, scalability, evaluation, and automation, data acquirement and quality improvement, and allowing interaction. The findings of this study help better understand what and how Web intelligence can be applied to promote healthcare procedures and clinical outcomes. This provides important insights into the effective use of Web intelligence to support informatics-enabled brain studies.

**Supplementary Information:**

The online version contains supplementary material available at 10.1186/s40708-022-00153-9.

## Introduction

Brain informatics, as brain sciences in the era of Web intelligence-centered information technology [1, 2], focuses on a systematic methodology to study human information processing mechanisms by using informatics [3]. The relationship between brain informatics and Web intelligence is significant [4]. For one thing, Web intelligence-centered information technologies can be applied to support brain science studies. For example, the wisdom Web and knowledge grids allow high-speed, large-scale analysis, simulation, and computation as well as novel ways to share research data and scientific achievements. For another thing, informatics-empowered brain studies can considerably broaden the spectrum of theories and models of brain sciences and provide new insights into developing human-level intelligence on the wisdom Web and knowledge grids. In this sense, to promote the study of brain informatics, there is an urgent research need to study and promote the field of Web intelligence. World Wide Web is an omnipresent system and vast data production and consumption platform where massive data are transmitted between diverse devices worldwide under divergent distributed settings. Web Intelligence, particularly Wisdom Web of Things, offers a social-cyber-physical space where big data are adopted to link humans, computers, and things [5–7]. As a leading field in artificial intelligence (AI), Web Intelligence resolves open issues to deepen the understanding of connectivities, phenomena, and developments in exploiting the power of human brains and digital networks. Web intelligence explores “the fundamental roles and practical impacts of AI and advanced information technologies on the next generation of Web-based products and services (p. 29) [8]”. It revolutionizes how information is stored, managed, shared, and implemented electronically, virtually, globally, standardized, and personalizedly [9]. Aiming at realizing the multidisciplinary balance between theoretical and methodological advances related to collective intelligence, data science, human-centered AI, autonomous agents, and multiagent systems, Web intelligence has the potentials to enhance our understanding of computational, cognitive, physical, and social foundations of the future Web, and promote intelligent technologies’ advances and applications.

Web intelligence commonly involves emergent topics associated with some broad, general fields. For instance, Web intelligence scholars show interest in data manipulation, ways to create distributed intelligence, agent self-organization, learning, and adaptation, agent-driven knowledge discovery and management, autonomic computing, Web security, semantic Web, Web services, and social intelligence [8]. When it comes to big brain data computing, the joint use of Web intelligence and brain informatics promotes human-level Web intelligence reality by advancing how we analyze and understand data, information, knowledge, wisdom, and how they interrelate [10]. Such interdisciplinary nature facilitates the application of brain informatics in brain intelligence, brain health, and brain Internet [11, 12].

Web intelligence has great potentials to contribute to diverse domains, for example, e-learning, e-governments, e-communities, and particularly e-health, which is experiencing a significant revolution. Web intelligence’s features such as ontologies, adaptivity, personalization, and agents have long attracted e-health researchers. Diverse Web applications and systems based on AI are developed. For instance, natural language processing (NLP) and automatic information retrieval retrieve and analyze Web blogs containing healthcare themes to promote the clinical research community’s understanding of feelings and emotions [13]. Web mining uses data mining technologies to automatically identify and extract information from large volumes of patients’ Web documents and is a low-cost and noninvasive approach for healthcare personalization [14]. Clustering and visualization based on machine learning algorithms allow data capturing, sharing, analysis, and decision making for effective, real-time disease surveillance.

In literature analysis, bibliometric analysis and systematic analysis are commonly adopted [15]. This study adopts systematic review methodologies because they have advantages over bibliometric approaches by allowing a more profound and fine-grained understanding of a research area [16, 17]. In contrast, although bibliometric analysis can quickly tackle large literature data, it usually fails to provide an in-depth investigation [18]. Our study is the first in-depth review that systematically examines the role of Web intelligence applications for human health. By examining 79 empirical studies in which 5941 health websites and 1329 Web pages were searched systematically for specific health information, Eysenbach et al. [19] summarized 408 evaluation outcomes based on 86 quality criteria. Barros et al. [20] systematically reviewed research findings in relation to Internet-based sources’ application for public health surveillance. Eysenbach et al. [19]’s and Barros et al. [20]’s reviews, however, focused on studies assessing customers’ Web health information quality and Internet-based sources for public health surveillance, respectively, rather than Web intelligence for human health. To provide a general picture showing how Web intelligence assists human health, a review comprehensively and systematically analyzing literature that proposes and evaluates Web intelligence applications and systems for human health appears essential.

To that end, this review aims at identifying and summarizing the extant literature on Web intelligence applications used for human health. To be specific, the included literature is synthesized from the perspectives of: (1) study characteristics, (2) AI applications, (3) clinical tasks, (4) scopes of Web intelligence, and (5) performance evaluation. We also provide suggestions for future research on Web intelligence for human health. It contributes to deepening the understanding of the benefits and challenges concerning Web intelligence for human health, for instance, by promoting healthcare procedures and clinical outcomes. This also contributes to the field of brain informatics by offering important insights into the effective and efficient applications of Web intelligence-centered information technologies to support high-speed, large-scale analysis, simulation, and computation, as well as novel ways to share research data and scientific discoveries in informatics-empowered brain studies.

## Data and methods

**Fig. 1:**
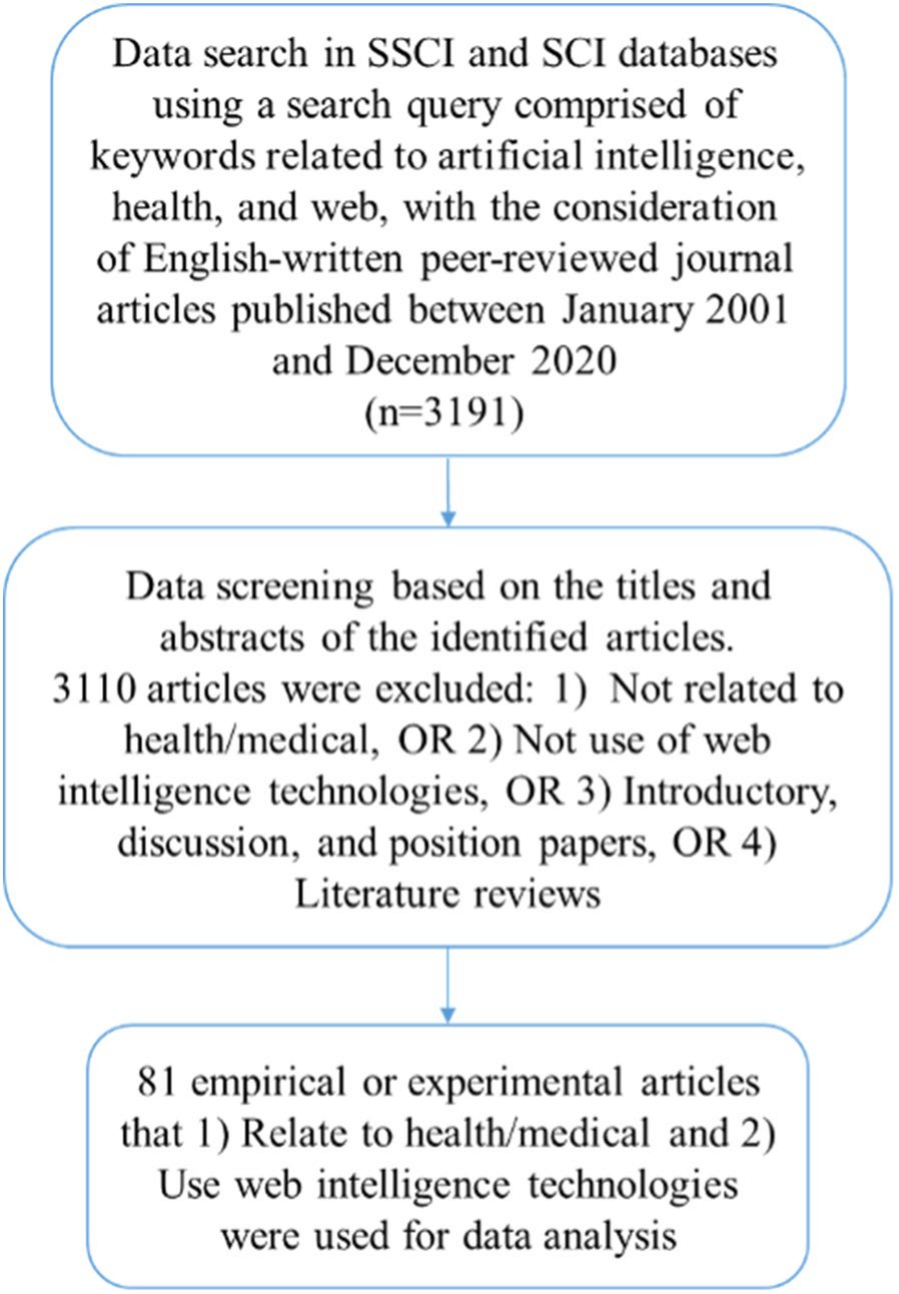
Data search and screening

The data search and screening are illustrated in Fig. 1.

### Search strategies

We collected data from Science Citation Index (SCI) and Social Sciences Citation Index (SSCI) databases since the quality of a journal article from SCI/SSCI journals is typically guaranteed, as suggested by previous studies [21, 22]. On 24 January 2021, we searched the two databases to identify research articles on Web intelligence applications for human health. We further limited the search results to English-written journal articles published from January 2001 to December 2020.

We adopted three sets of keywords to match terms in the title, abstract, or keyword of a publication. The first set was AI-related terms, which were determined based on the search terms used in [23]. In addition to broadly encompassing terms such as “artificial intelligence”, “machine learning”, and “deep learning”, we also included specific AI techniques, such as “neural network*”, “support vector machine*”, “decision tree*”, “random forest”, “neural network*”, and “artificial neural network”. The second set of keywords was related to health. Previous studies mostly used “health*” as a search term to retrieve articles focusing on health or healthcare. For example, Jalali et al. [24] used “Health* and Guo et al. [25] adopted “electronic health record”, “health”, “healthcare”, “medicine”, “mental health”, and “behavior health”. In this study, in addition to adopting broad terms “health*” and “medicine”, we also considered “medical”, “nursing”, “smart care”, and “elder care”, as they also related closely to health and healthcare. The third set was Web-related keywords, which were determined by referring to Eysenbach et al. [19]. Specifically, in addition to including “web”, “www”, and “Internet” following Eysenbach et al., we also considered “web-based” OR “website*”. However, to avoid noise, we excluded “Internet of things” as it describes physical objects that are embedded with sensors, processing ability, software, and other technologies, which are not within the scope of this study. The specific search strategy is listed in Additional file 1: Table S1. A total of 3191 articles were obtained.

### Eligibility criteria

We downloaded the metadata information (i.e., titles, years of publication, authors and their institutions, and abstracts) of all the identified articles. Two domain experts individually examined the title and abstract of an identified article to decide its eligibility based on the criteria listed in Table 1. Disagreements were addressed through discussion between the experts until an agreement was reached [26]. When we decided whether a paper should be included, we began from the first exclusion criterion (i.e., not related to health/medical) and excluded it directly in case it was. Then, we checked whether it mentioned the use of Web intelligence technologies. After confirming that the paper was related to the use of Web intelligence technologies for human health, we checked whether it was original research and omitted those that were reviews, introductory, discussion, and position papers. Totally, 81 articles remained, the full texts of which were downloaded.

**Table 1:** Inclusion and exclusion criteria

Types	Criteria	Note
Inclusion criteria	Related to health/medical	An included article should meet both criteria
	Use of Web intelligence technologies	
Exclusion criteria	Not related to health/medical	An excluded article should meet one of the criteria
	Without the use of Web intelligence technologies	
	Introductory, discussion, and position papers	
	Literature reviews	

### Coding scheme

A coding scheme was constructed to identify information from the 81 articles in terms of: (1) study characteristics, (2) clinical tasks, (3) AI applications, (4) scopes of Web intelligence, and (5) evaluation outcomes (Table 2). The complete coding results are listed in Additional file 1: Table S2.

**Table 2:** Coding scheme

Elements	Codes
Study characteristics	Year, journal, Web of Science (WOS) category, country/region, institution
Health	Study design, clinical tasks
AI applications	Types of AI technologies
Web intelligence	Scopes of Web intelligence
Evaluation outcomes	Performance evaluation matrix

## Results and discussion

### Study characteristics

**Fig. 2:**
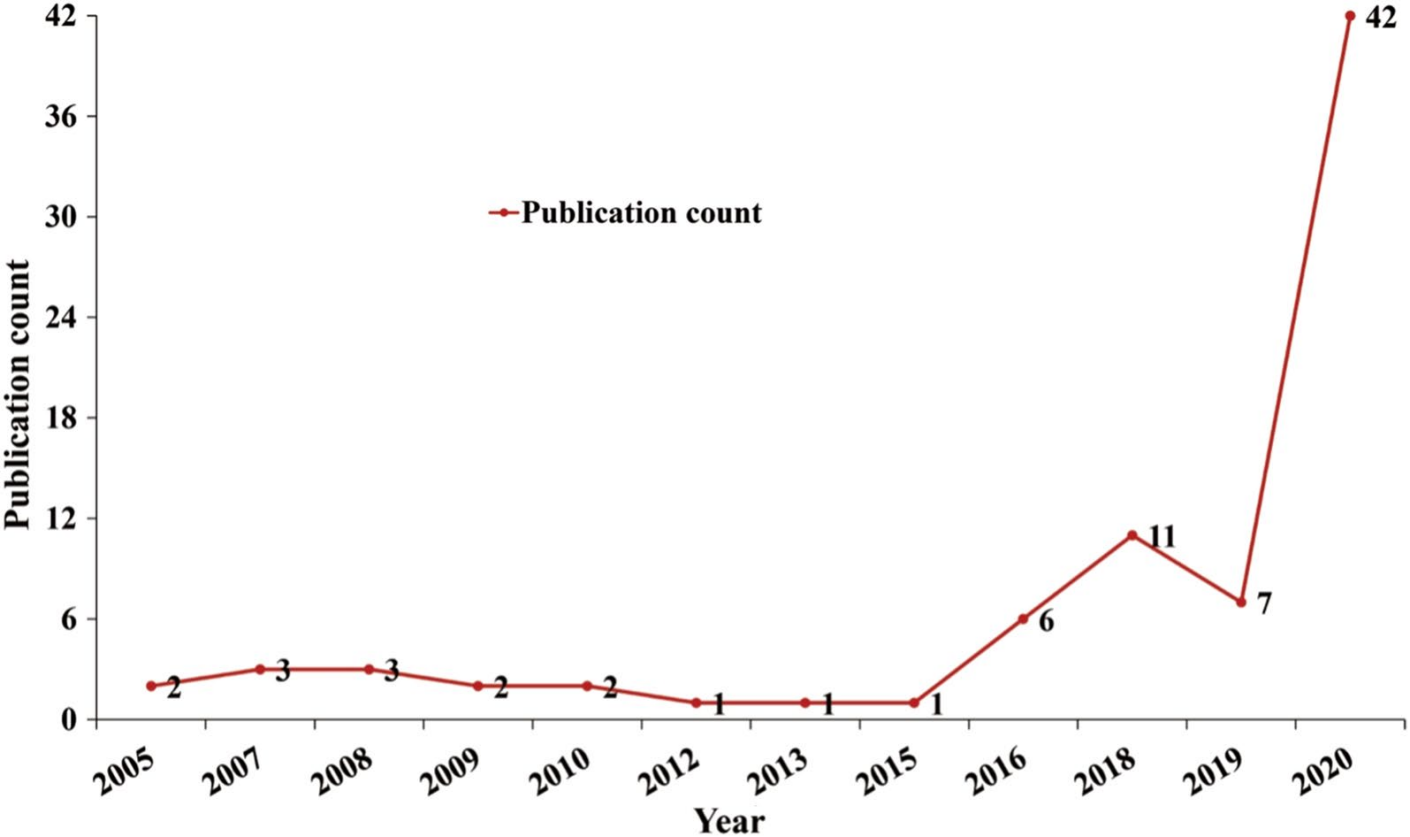
Year of publication

Figure 2 shows that the number of the included studies has increased year by year, indicating increasing activeness in research activities associated with Web intelligence for human health. Scholars majorly started examining Web intelligence for human health in 2005, with two articles available. Specifically, Colombet et al. [27] focused on the use of decision trees for knowledge specification in Web-enriched decision support systems that allowed users to personalizedly evaluate risks and receive recommendations based on their clinical profiles. Bellika and Hartvigsen [28] developed a Web-based intelligent oncological nurse advisor via information retrieval using neural networks. In the following years, the annual number was around one to three. Since 2015, research output on Web intelligence for human health increased constantly and dramatically, reaching a peak in 2020 with 42 articles. The top ten cited studies are presented in Table 3. Li et al. [29]’s work with 141 citations focuses on the development of a support vector machine (SVM)-Prot web-server for predicting protein functional families from protein sequences regardless of similarities. With 109 citations, Abacha et al. [30]’s work about proposing a medical question answering system based on NLP and semantic Web is the second most cited. The third paper with 61 citations was contributed by Graber and Mathew [31], who developed a Web-oriented clinical decision support system for facilitating medical diagnosis.

**Table 3:** Top ten cited studies

Study	Publication source	PY	TC
Li et al. [29]	BMC Medical Informatics and Decision Making	2010	141
Abacha et al. [30]	PLOS ONE	2016	109
Graber and Mathew [31]	Information Processing & Management	2015	61
Rau et al. [32]	Journal of General Internal Medicine	2008	53
Huang and Chen [33]	Computer Methods and Programs in Biomedicine	2016	40
Falkman et al. [34]	Expert Systems with Applications	2007	26
Forster et al. [35]	Journal of Medical Internet Research	2008	23
Zheng et al. [36]	Journal of Medical Internet Research	2016	19
Konovalov et al. [13]	JMIR Medical Informatics	2016	18
Yu et al. [37]	Journal of Medical Internet Research	2010	17

PY, year of publication; TC, citations counted up to 24 January 2021 in WoS

**Fig. 3:**
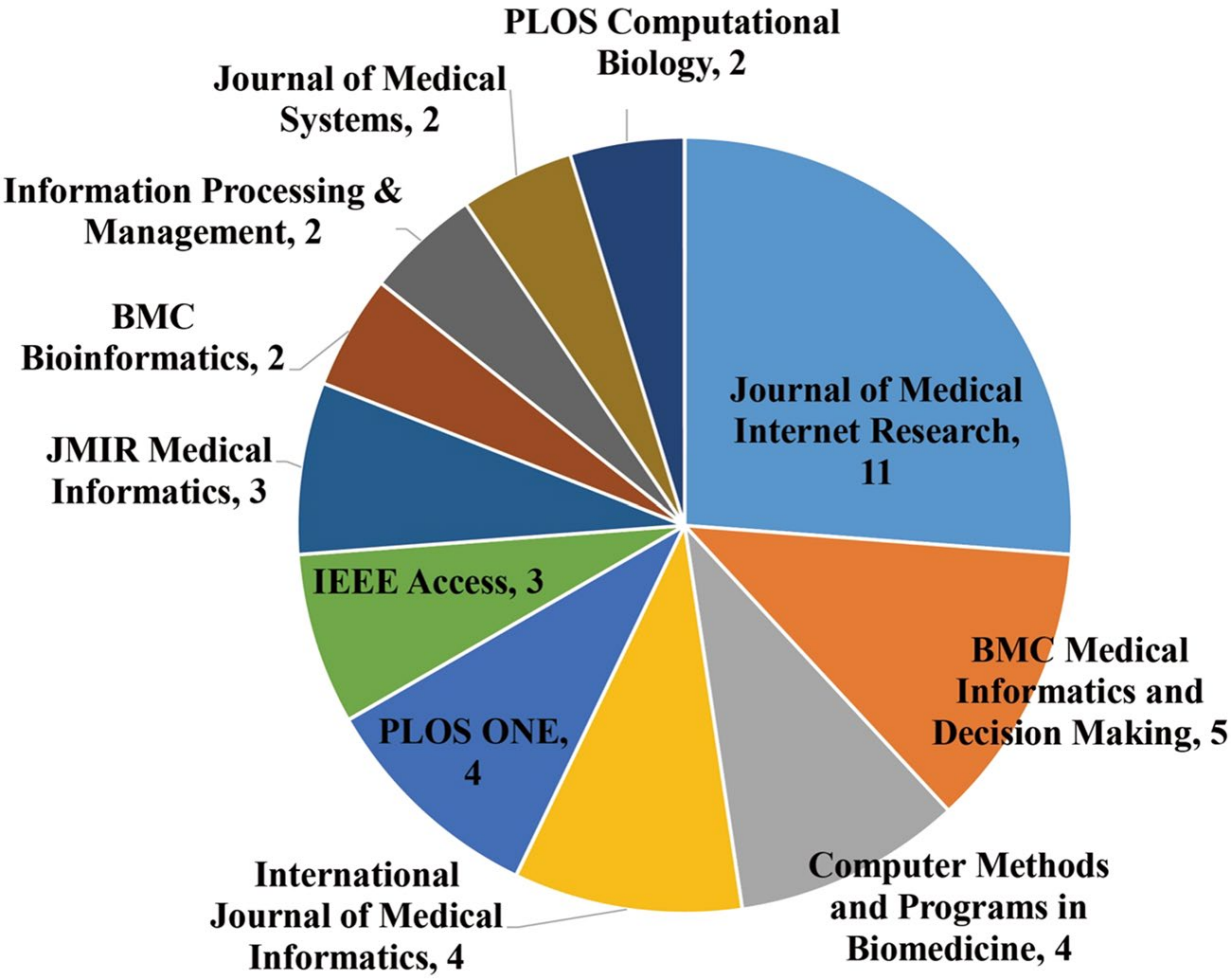
Top productive journals

The 81 articles were distributed in 50 journals. The top ones ranked by productivity (Fig. 3) accounted for 52% of the total articles. The top productive ones were *Journal of Medical Internet Research* (11 articles) and *BMC Medical Informatics and Decision Making* (5 articles). The first two published research about digital medicine and healthcare, and the last focused on designing, developing, applying, and assessing healthcare information technologies and their effectiveness for decision making. Other important journals included *Computer Methods and Programs in Biomedicine*, *International Journal of Medical Informatics*, and *PLOS ONE*, each with four articles. Among the listed journals, six were related to medical informatics (i.e., *Journal of Medical Internet Research*, *BMC Medical Informatics and Decision Making*, *Computer Methods and Programs in Biomedicine*, *International Journal of Medical Informatics*, *JMIR Medical Informatics*, and *Journal of Medical Systems*) and three were about computer science, information system (i.e., *International Journal of Medical Informatics*, *IEEE Access*, and *Information Processing & Management*).

**Fig. 4:**
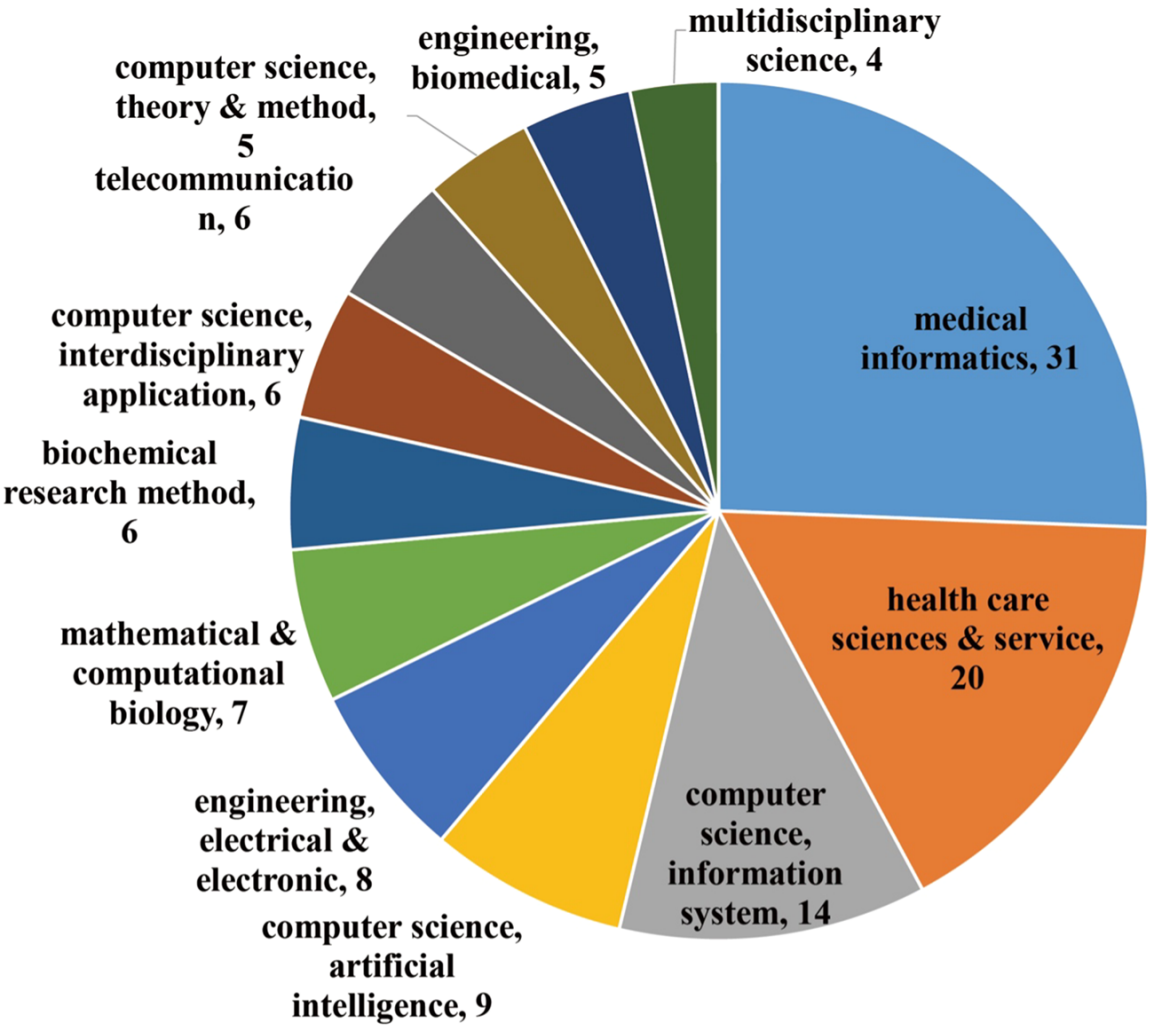
Top WoS subjects

In subject analysis, 36 WoS subjects were identified, with the top 12 being listed in Fig. 4. *Medical informatics* was ranked at first place, occupying about 38% of the corpus. The *health care sciences & service* was ranked second, accounting for 25%. Other important subjects included *computer science, information system* (14 articles), *computer science, artificial intelligence* (9 articles), and *engineering, electrical and electronics* (8 articles).

**Fig. 5:**
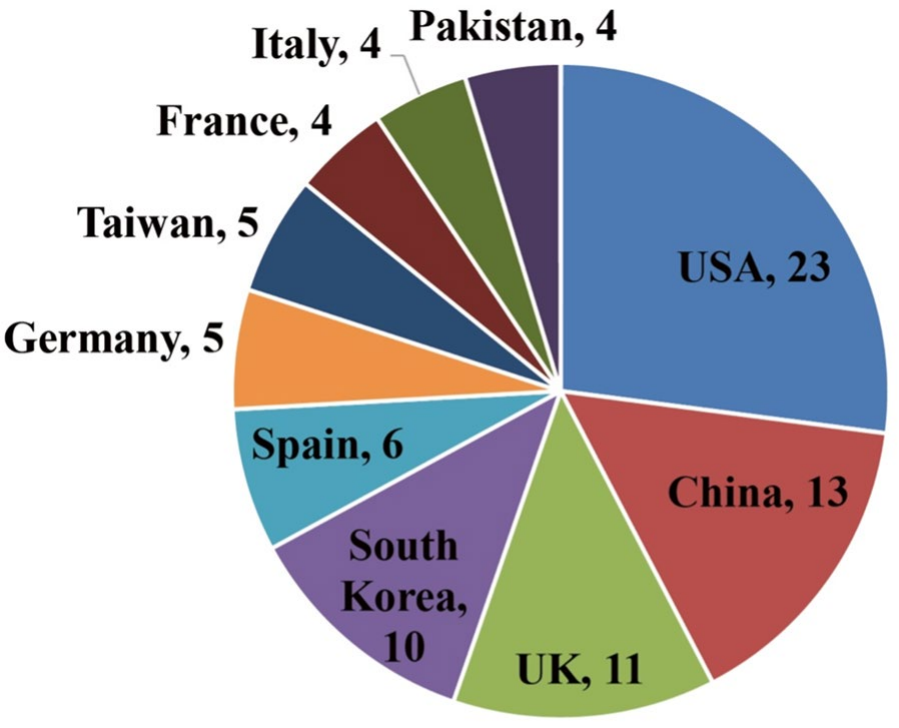
Top countries/regions

**Fig. 6:**
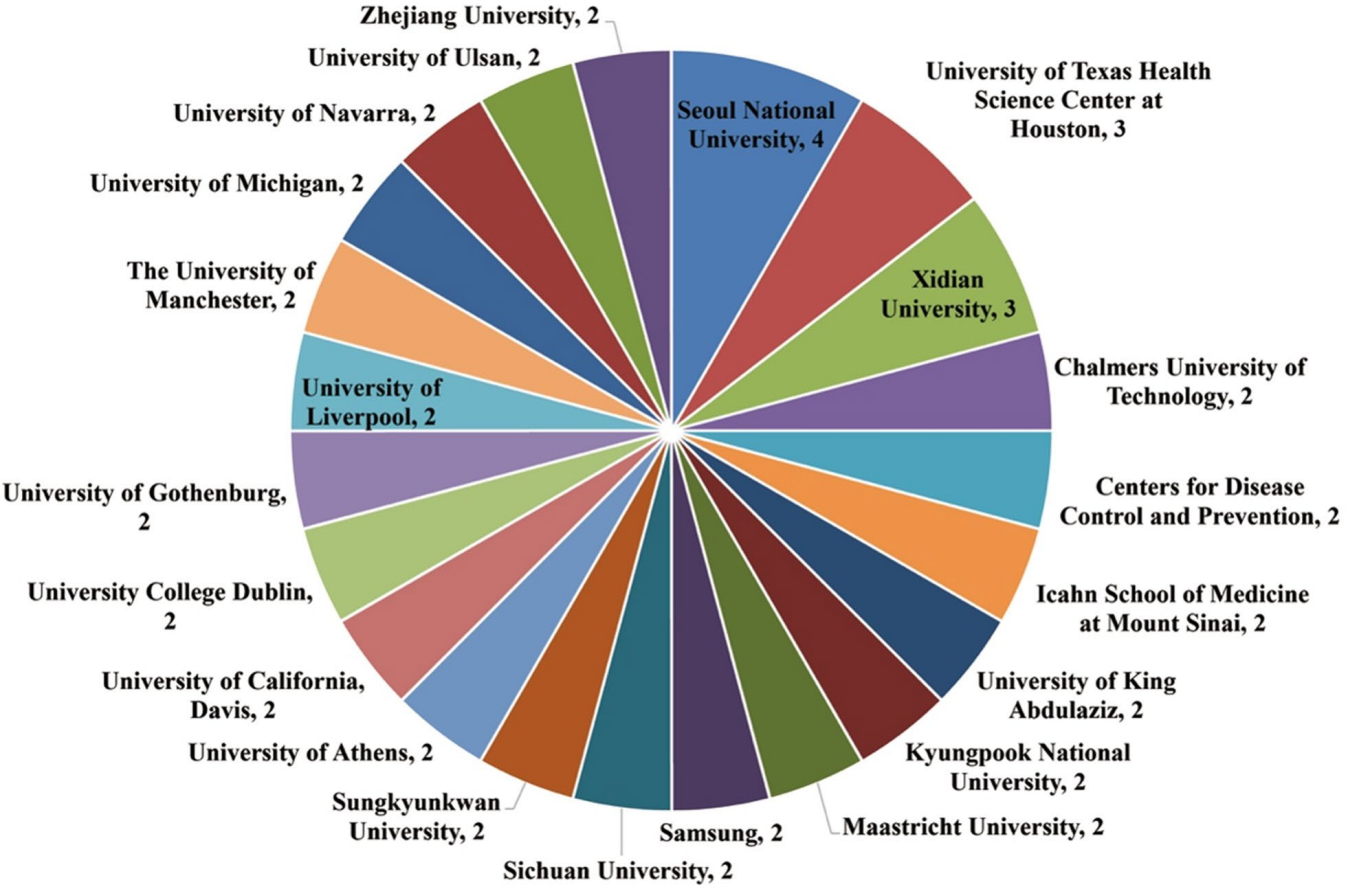
Top institutions

There were 31 countries/regions and 221 institutions. Figure 5 presents the top ten countries/regions ranked by productivity, indicating the active role of scholars from the USA, China, the UK, and South Korea in Web intelligence-assisted human health research. Figure 6 presents the top 22 institutions ranked by productivity. The top three productive institutions were Seoul National University (4 articles), the University of Texas Health Science Center at Houston (3 articles), and Xidian University (3 articles).

**Fig. 7:**
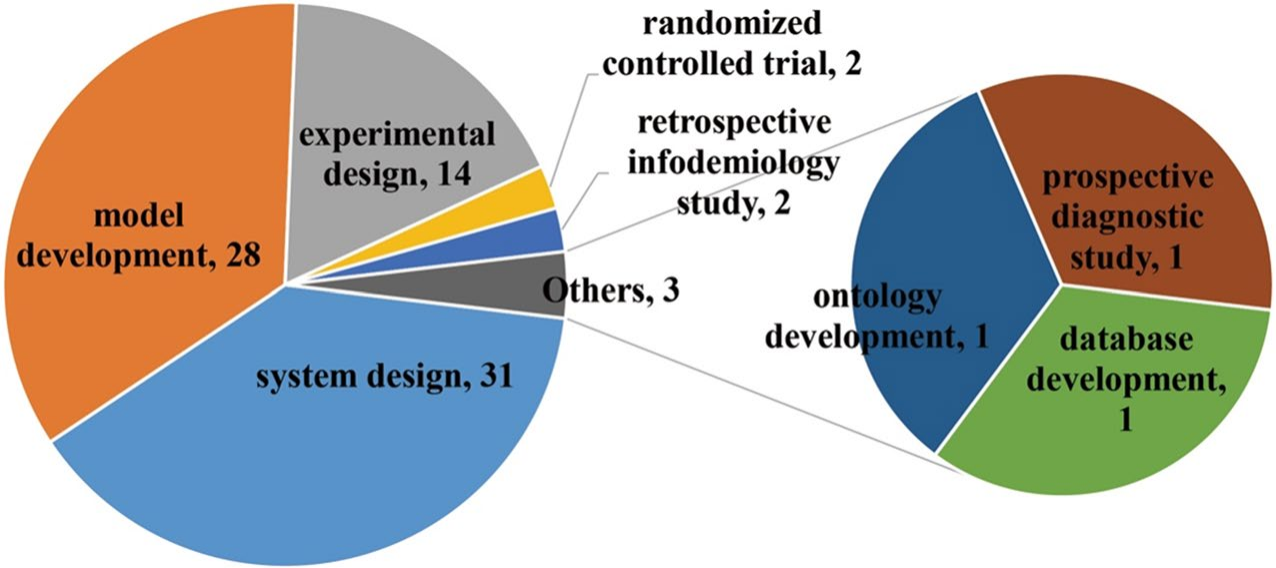
Distribution of study design

Figure 7 shows the distribution of the study design. There were 31 studies concerning system design, 28 about model development, 14 about experimental design, 2 were randomized controlled trials, 2 were retrospective infodemiology studies, 1 about ontology development, 1 about database development, and 1 was a prospective diagnostic study.

### AI applications

**Fig. 8:**
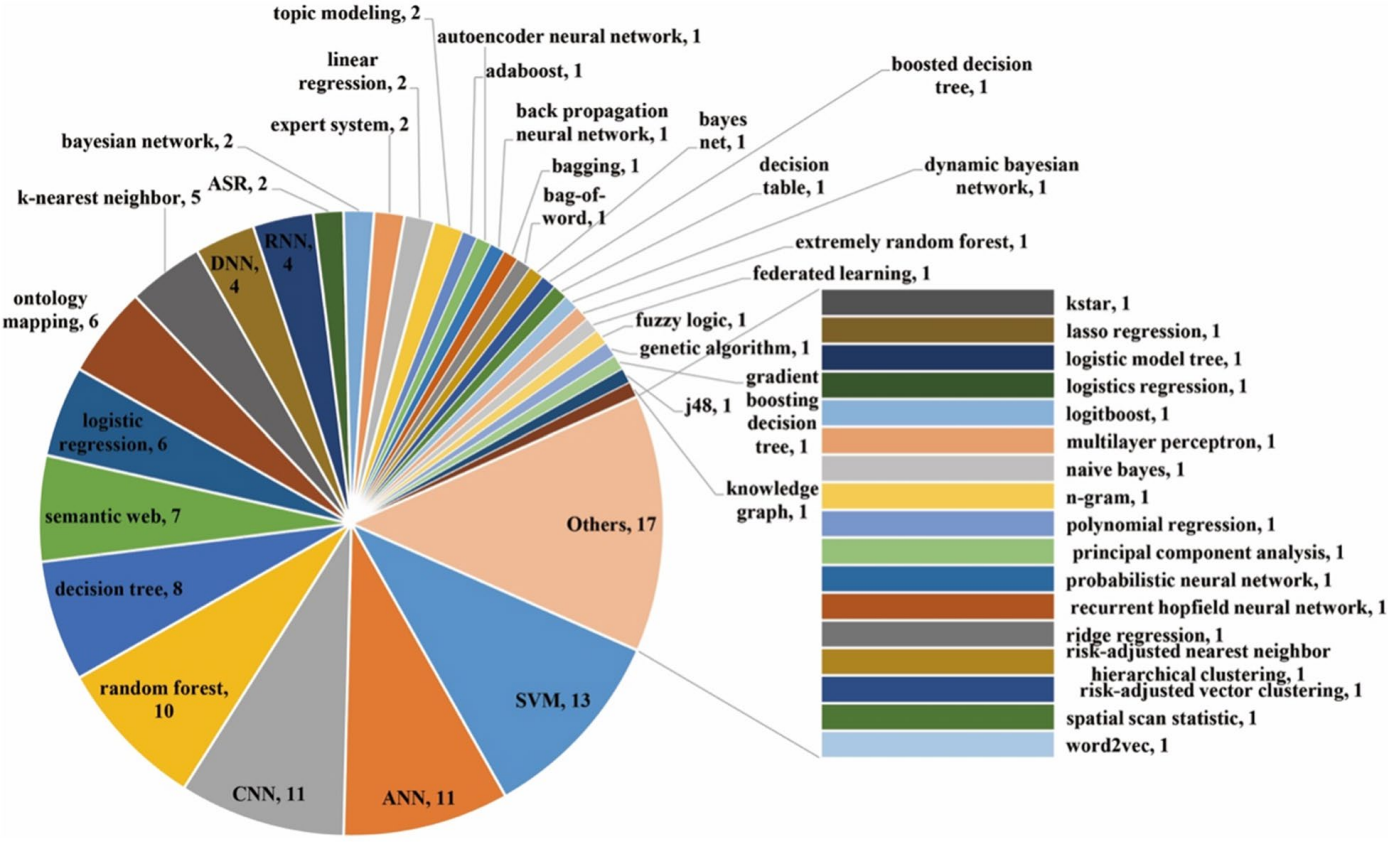
Distribution of AI technologies

**Fig. 9:**
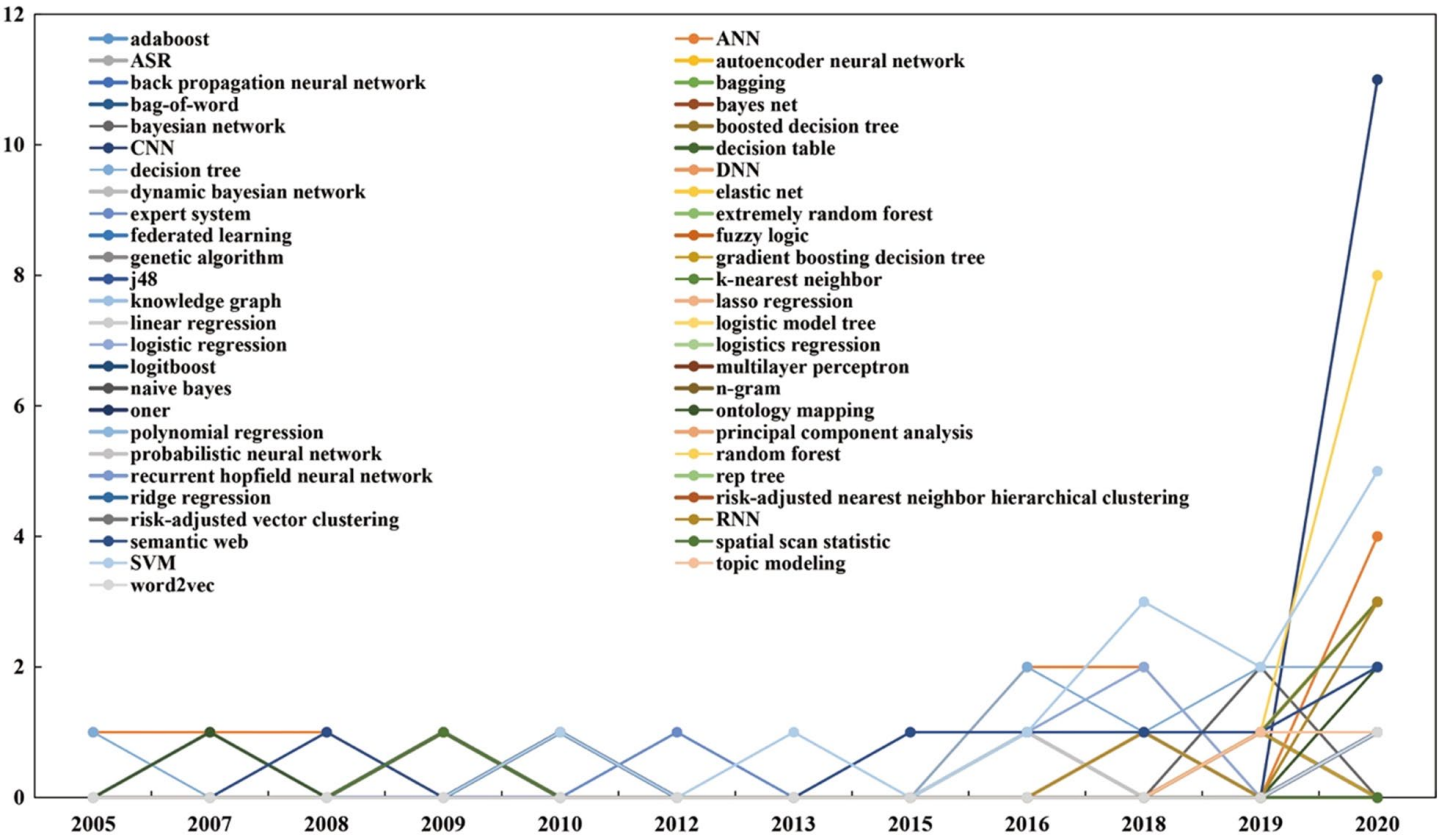
Distribution of AI technologies by year

Figure 8 shows, among the 81 studies, SVMs were the most popular (*n* = 13), followed by artificial neural networks (ANNs) (*n* = 11), convolutional neural networks (CNNs) (*n* = 11), random forests (*n* = 10), decision trees (*n* = 8), and semantic Web (*n* = 7). Other important AI technologies included logistic regression (*n* = 6), ontology mapping (*n* = 6), k-nearest neighbors (*n* = 5), deep neural networks (DNNs) (*n* = 4), recurrent neural networks (RNNs) (*n *= 4), automatic speech recognition) (*n* = 2), Bayesian network (*n* = 2), expert system (*n* = 2), linear regression (*n* = 2), and topic modeling (*n* = 2). From an evolution perspective (Fig. 9), most technologies showed a growing tendency in usage, especially random forests and CNNs. Some AI technologies (e.g., decision trees, expert systems, and ANNs) started to be employed at an early stage, while technologies like autoencoder neural networks, back-propagation neural networks, CNNs, DNNs, fuzzy logic, genetic algorithms, knowledge graphs, and RNNs received attention at later periods.

### Clinical tasks

**Fig. 10:**
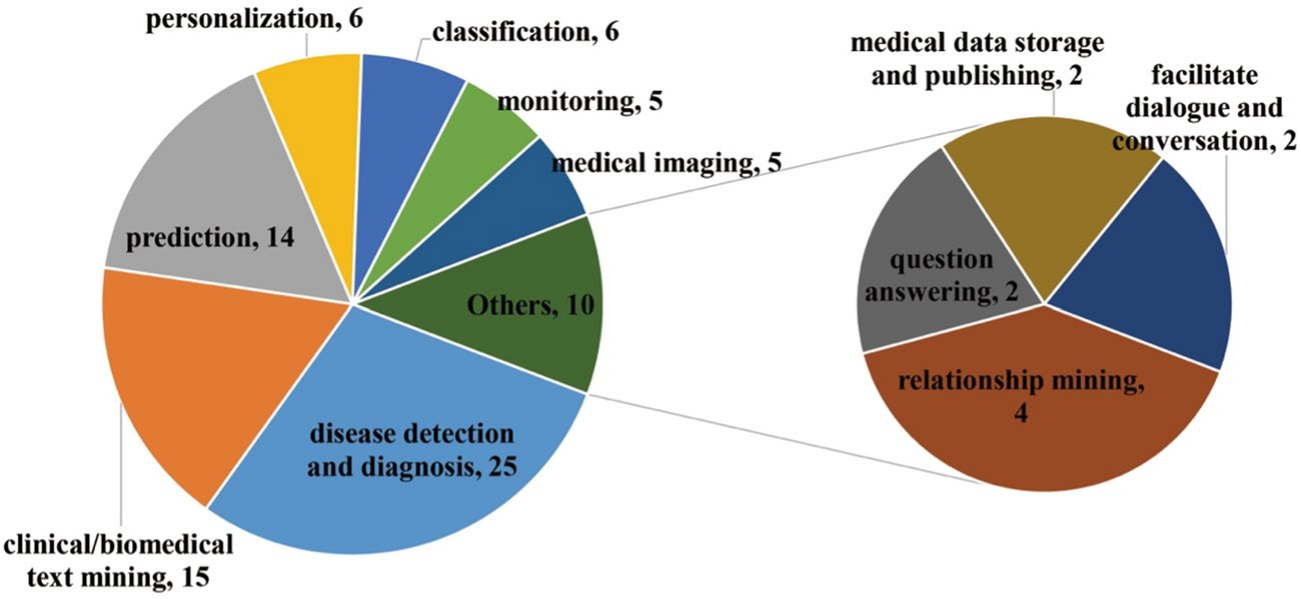
Distribution of clinical tasks

**Fig. 11:**
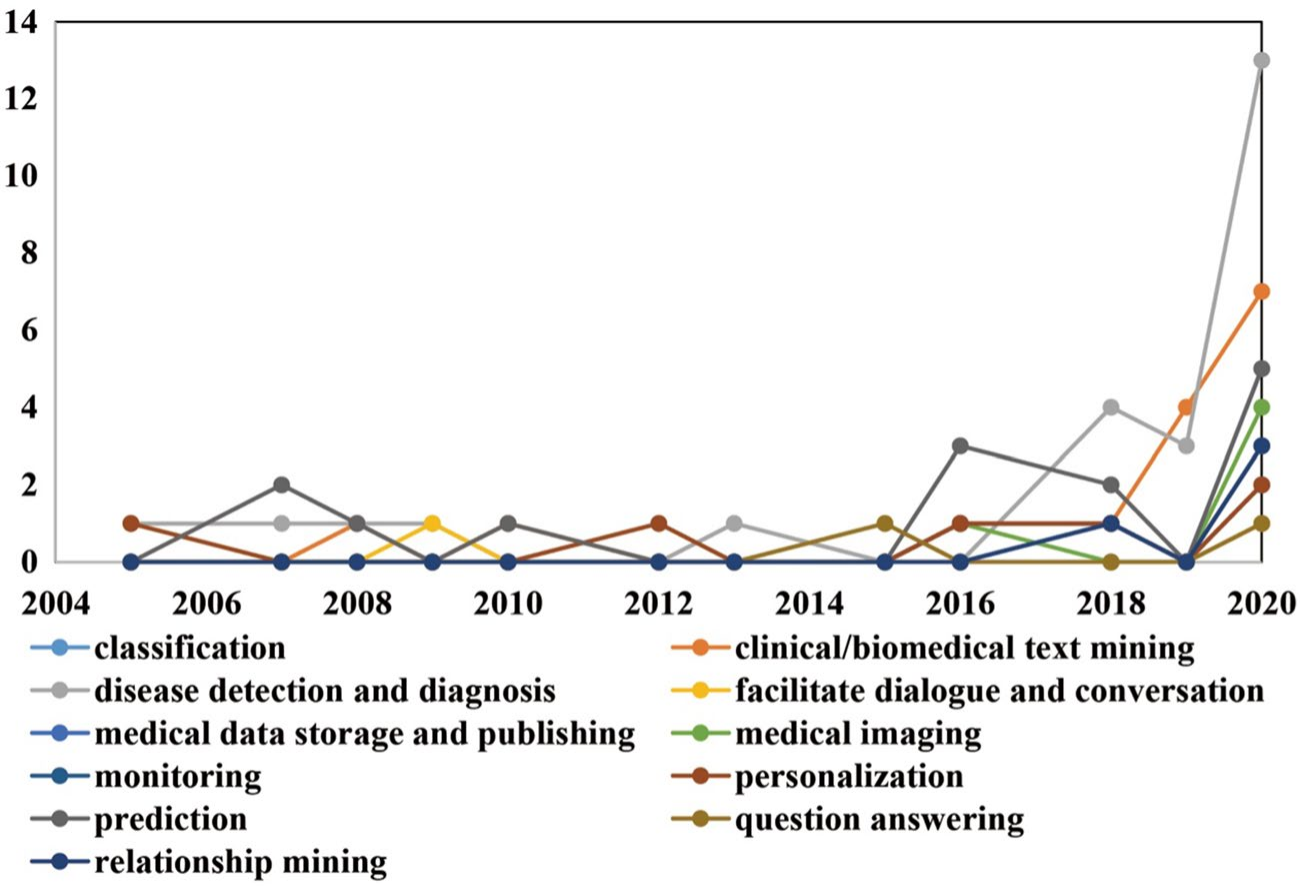
Distribution of clinical tasks by year

Figure 10 shows diverse Web intelligence technologies were mainly used for disease detection and diagnosis (*n* = 25), followed by clinical/biomedical text mining (*n* = 15) and prediction (*n* = 14). Other important tasks included personalization (*n* = 6), classification (*n* = 6), monitoring (*n* = 5), medical imaging (*n* = 5), relationship mining (*n* = 4), question answering (*n* = 2), medical data storage and publishing (*n* = 2), and facilitating dialog and conversation (*n* = 2). From an evolution perspective (Fig. 11), with the passage of time almost all tasks were increasingly considered and facilitated by diverse Web intelligence technologies, especially disease detection and diagnosis and clinical/biomedical text mining. Some tasks (e.g., disease detection and diagnosis, personalization, and prediction) started to gain popularity at an early stage, while issues like classification, medical data storage and publishing, medical imaging, monitoring, question answering, and relationship mining were utilized at a later period.

### Scopes of Web intelligence

**Fig. 12:**
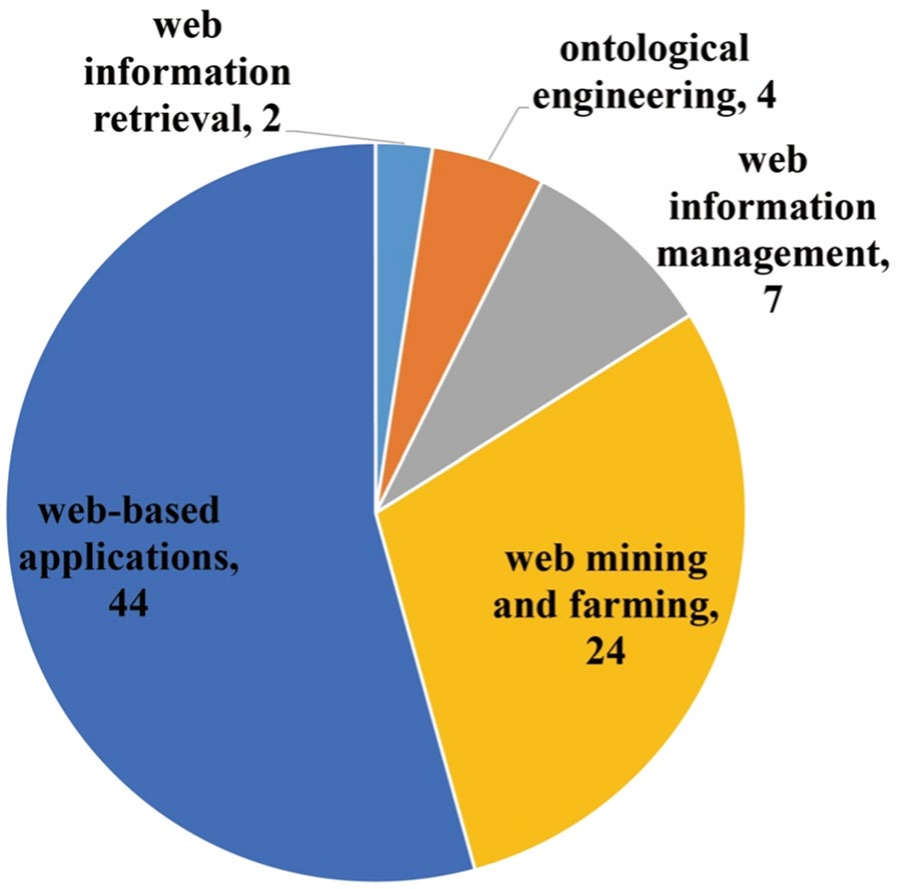
Distribution of scopes of Web intelligence

**Fig. 13:**
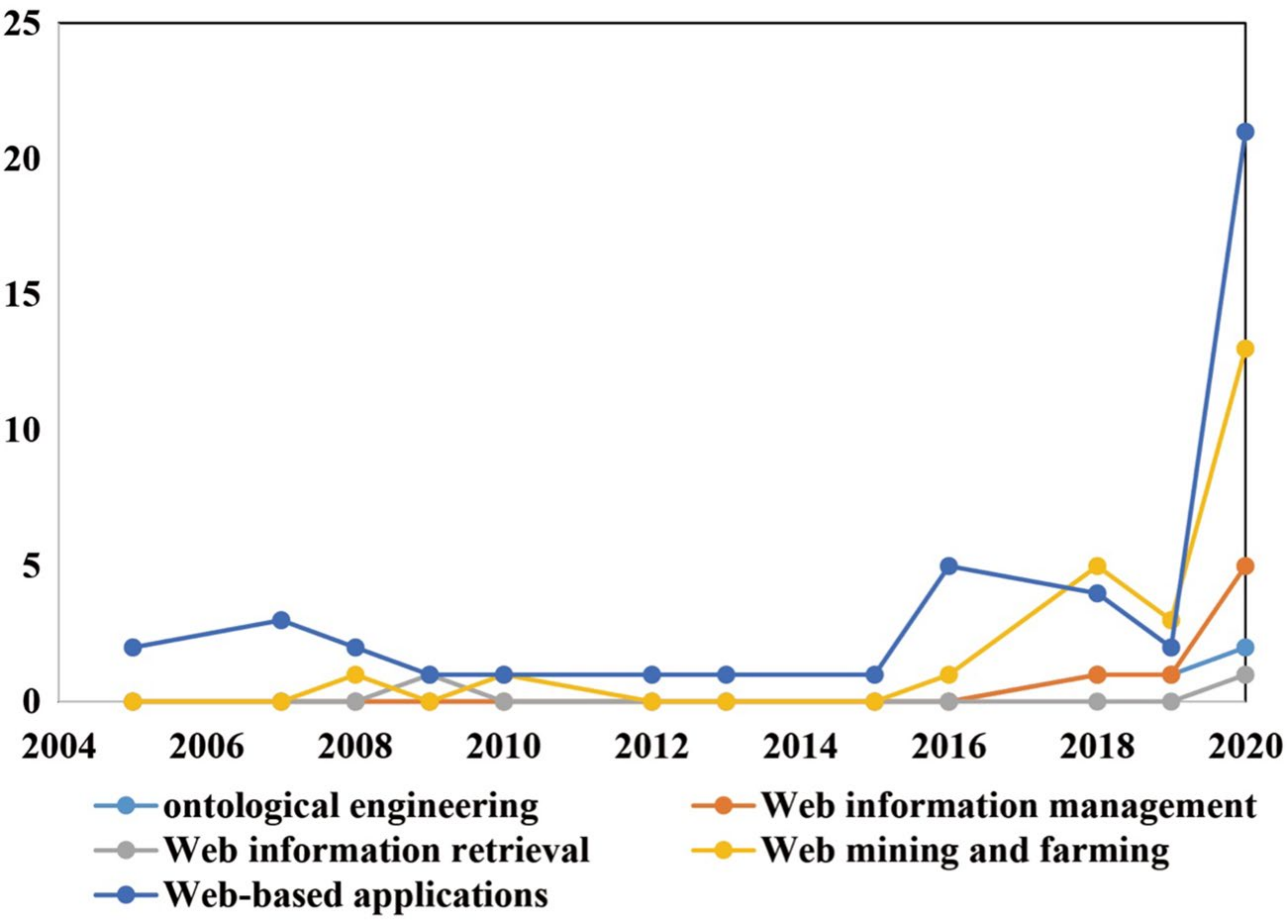
Distribution of scopes of Web intelligence by year

Inspired by Zhong et al. [38], we categorized the scopes of Web intelligence (Fig. 12) in the 81 articles into Web-based applications (*n* = 44), Web mining and farming (*n* = 24), Web information management (*n* = 7), ontological engineering (*n* = 4), and Web information retrieval (*n* = 2). From an evolution perspective (Fig. 13), with the passage of time most of the scopes were increasingly concerned with academia, especially Web-based applications and Web mining and farming. Some scopes (e.g., Web-based applications) started to gain popularity at an early stage, whereas scopes such as ontological engineering and Web information management received attention at later periods.

### Performance evaluation

**Fig. 14:**
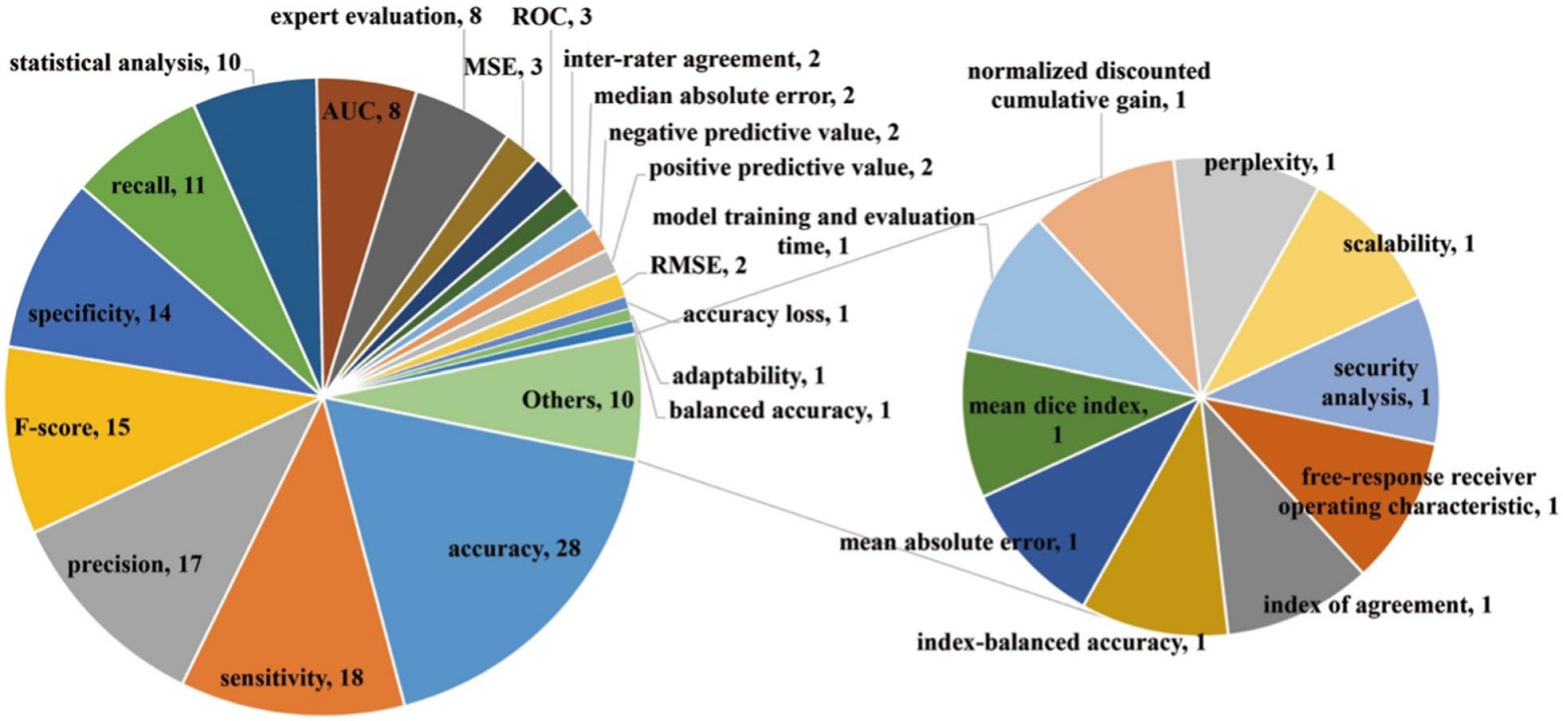
Distribution of performance evaluation indicators

In terms of performance evaluation of Web intelligence applications for human health (Fig. 14), commonly used metrics included accuracy (*n* = 28), sensitivity (*n* = 18), precision (*n* = 17), F-score (*n* = 15), specificity (*n* = 14), and recall (*n* = 11). Ten studies adopted statistical analysis mythologies. For example, Lakshmi et al. [39] adopted correlation, entropy, and histogram analyses to validate the statistical resistivity of a Hopfield neural network (HNN)-driven image-dependent encryption framework for storing medical images on the cloud. In Ref. [40], the reliability of a trained partial logistic ANNs for predicting risk of death in patients with colorectal cancers was measured by Kaplan–Meier observed survival analysis. Other performance indicators included area under the curve (AUC) (*n* = 8), expert evaluation (*n* = 8), mean-square error (MSE) (*n* = 3), receiver operating characteristic curve (*n* = 3), inter-rater agreement (*n* = 2), median absolute error (*n* = 2), negative predictive value (*n* = 2), positive predictive value (*n* = 2), and root MSE (RMSE) (*n* = 2).

### Relationship between AI, tasks, and scopes of Web intelligence

**Fig. 15:**
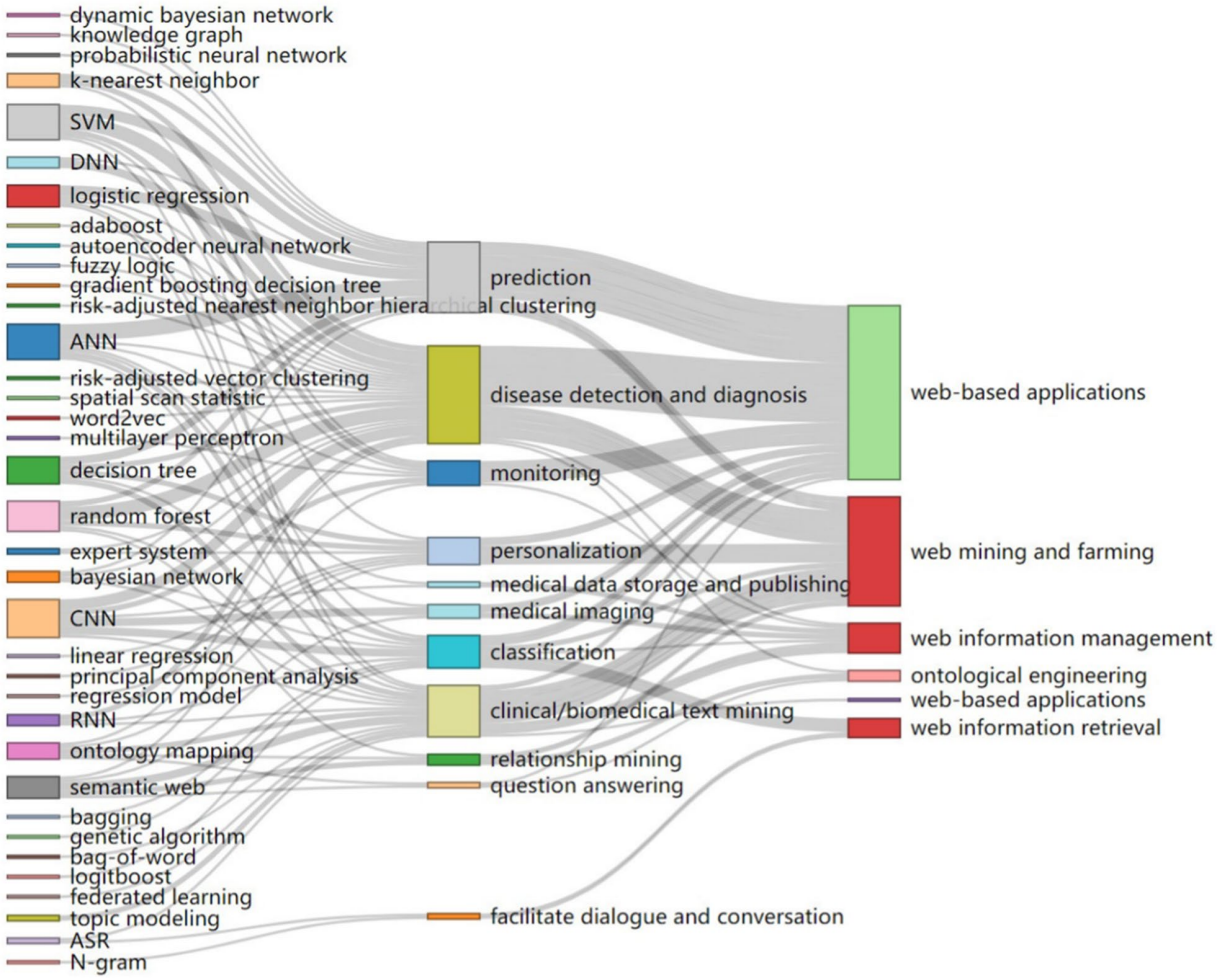
Relationship between AI, tasks, and Web intelligence scopes (downloading interactive graphics via https://drive.google.com/file/d/1kkA0oo8VZ4DhyIiFliW7S59pUKjW_hRI/view?usp=sharing)

Figure 15 visualizes the relationship between AI, tasks, and Web intelligence scopes.

#### Disease detection and diagnosis

In terms of disease detection and diagnosis, AI algorithms have been widely integrated into Web-based applications and used for Web mining. For example, Sun et al. [41] developed an SVM-based Web system for improving ion channel-targeted conotoxin prediction. Shah et al. [42] utilized SVMs, random forests, and K-nearest neighbors to monitor the physical activities of patients with epileptic seizures by treating wireless devices as sensors in medical cyber-physical systems. Hu et al. [43] utilized a flexible tree-driven principled variable selection approach to facilitate the identification and ranking of the significance of determinants of high medical expenses and their effects among patients with breast cancers.

Second, NLP technologies are commonly integrated into Web-based medical and clinical diagnosis and support systems for disease detection and diagnosis. For example, in Ref. [44], word embedding technologies were combined to automatically detect diseases with the basis of social media posts and evaluated the technologies using Word2Vec with skip-gram. Bala et al. [45] developed a Web tool to allow users to find radiology reports “documenting the presence of a newly discovered adrenal incidentaloma (p. 606)” in real time. Huang and Chen [33] developed an intelligent disease diagnosis system for efficiently eliciting expert knowledge and constructing medical ontologies. In a new, second-generation Web clinical decision support system [31], NLP and search algorithms were used to compare terms entered by the clinician to those adopted in referred libraries.

Thrid, CNNs have been applied in disease detection and diagnosis for myosteatosis assessment [46], emotion and pain recognition [47], and oral hygiene management [48]. Specifically, a Web toolkit [46] “generated a muscle quality map by categorizing muscle components and evaluated the feasibility of automated quantitative measurements of the skeletal muscle on computed tomography images to assess normal-attenuation muscle and myosteatosis (p. 1)” using a pre-developed CNN-based deep learning algorithm. Pandit et al. [47] developed a shallowest-possible CNN algorithm to forecast emotions in real time from real life, noisy, laggy videos on the Internet. In [48], a light-induced fluorescence-based system visually identified dental plaques and presented the location of the dental plaques on oral images using Mask region-based CNNs.

Furthermore, DNNs have played an important role in Web-based applications for disease detection and diagnosis (e.g., teledermatology [49], lung cancer [50], and phenotyping psychiatric disorders [51]). Specifically, Muñoz‐López et al. [49] assessed DNN’s performance for diagnosing skin diseases based on patients’ submitted photographs of one or more skin conditions acquired using a smartphone prior to or during a teledermatology evaluation. A Web lung cancer annotation tool [50] annotated lung nodules regions to facilitate automatic lung nodule detection. Specifically, “when the annotators found a lung nodule on the computed tomography images, they could simply use the mouse to draw a rectangle around it, the location and size of the corresponding rectangle would be recorded as the label for lung nodules detection task (p. 199)”. An AI-integrated Web system [51] diagnosed schizophrenia under the guidance of explainable DNNs with three-dimensional visualization of subjects’ structural brain imaging data.

Additionally, there are other AI technologies that have been adopted for disease detection and diagnosis, including RNNs, fuzzy logic, expert systems, and autoencoder neural networks, for example, a passive device-free Fall detection system [52] “based on commodity WiFi framework for smart home was mainly composed of hardware platform and client application (p. 308)”, in which an RNN classified human motions and identified fall status. A Web expert system [53] diagnosed depression aided by fuzzy Delphi method through depression symptom weight and importance estimation. In [33], the Chinese Medical Diagnostic System adopted Web interface and expert system technologies as human experts for diagnosing digestive system diseases. Zeng et al. [54] virtually screened “compounds targeting precise groups of patients with cancer by using gene expression features (p. 728)” to find drugs that altered gene expression in such a way that they were likely to reverse the expression pattern of the disease.

#### Clinical/biomedical text mining

In clinical/biomedical text mining, AI technologies are commonly adopted in Web-based applications for Web information management and mining. For example, the comparison of SVMs, Bayesian networks, and decision trees for malicious crawler detection based on navigational behaviors [55] indicated the superiority of the SVMs in enhancing sensitive patients’ information security. Also, the extraction of proper features of log files increased SVMs’ performance. In Ref. [56], a sentence-ranking mechanism adopted random forests and multiple importance indicators for relevance measurement and sentence ranking. In Ref. [57], a privacy-preserving machine learning procedure (e.g., logistic regression) enabled training and evaluating “models on medical data from multiple sources while providing privacy protection for sensitive data (p. 1)”.

Second, NLP is also used in clinical/biomedical text mining for medical report construction [58], Web-based real-time case finding [36], and Web blog analysis [13]. Specifically, a Web system [58] developed based on the Google Web Speech API and Microsoft Bing Speech API generated medical reports via automatic speech recognition. A Web-based NLP diabetes case-finding method [36] developed based on structured and unstructured electronic medical records was proven effective in “identifying uncodified diabetes cases in real-time, leading to a significant improvement in diabetes case finding and a complete ascertainment of diagnoses of diabetes mellitus (p. 1)”. Konovalov et al. [13] used NLP to analyze military social media postings to develop a classifier via the manual selection of appropriate word unigrams as features for the extraction of combat exposure descriptions from Weblogs.

In clinical/biomedical text mining, scholars mainly focus on Web mining to build trustworthy and ecological health knowledge [59] and support distributed medical communities of practice [34]. Specifically, Arguello-Casteleiro et al. [59] adopted semantic Web and deep learning to scan massive “biomedical literature and clinical narratives to represent the meaning of biomedical and clinical terms by exploring how to turn information about diagnoses, prognoses, therapies, and other clinical concepts into computable knowledge using free-text data about human and animal health (p. 1)”. Falkman et al. [34] examined the communication patterns of distributed healthcare professionals to improve the structure of meetings and discussions.

Additionally, topic modeling and RNNs are commonly used for clinical/biomedical Web text mining. Schäfer et al. [60] identified “Web-based discussion topics associated with Gastrointestinal discomfort and its perceived factors in Web-based messages posted by users of French social media (p. 1)” using topic modeling to provide real-world evidence for caregivers. Chen et al. [61] employed topic modeling and visual analytics techniques to characterize textual content generated during Internet behavioral health interventions. In [62], an RNN-based semi-supervised learning algorithm exploited rich unlabeled Web corpus.

#### Prediction and classification

AI technologies have gained popularity in facilitating prediction in Web-based applications. For example, based on the analysis of time course of 46,170 virtual subjects who experienced varied lifestyle conditions using decision trees, random forests, Stolfi et al. [63] highlighted machine learning models’ effectiveness for predicting the synthetic dataset as a computationally cheaper “mathematical model to be implemented on mobile devices to allow self-assessment by informed and aware individuals (p. 508)”. A step-by-step analysis [64] indicated the feasibility of user journey data analysis in varied machine learning models to predict dropout in digital health interventions. ANNs are also widely adopted. For example, in [65], ANNs were proven to satisfactorily forecast acquired immune deficiency syndrome (AIDS) incidences based on search trend data from Baidu.com as input variables, with the officially reported authentic AIDS incidences and deaths as output variables, in spite of changes in search queries. Siristatidis et al. [66] developed an ANN-enhanced Web-based system to predict in vitro fertilization outcomes to help clinicians tailor personalized subfertile couple treatment, promote reproduction outcomes, and evaluate massive information rapidly and automatically for objective indication provision concerning the outcome of artificial reproductive cycles. In Ref. [67], an ANN-based Web-based decision-support tool “accurately predicted the no-show patients by using the variable set that was commonly selected by a genetic algorithm and simulated annealing (p. 1)”. In a combined multi-omics and time series data analysis scheme which recognized perturbed sub-pathways and regulatory mechanisms in drug response [68], “multi-omics potential mediator genes were chosen by embedding multi-omics data into gene-centric vector space using either a tensor decomposition or an autoencoder deep learning model (p. 3)”.

In terms of classification, scholars have focused on classification driven in Web-based applications. For example, a machine learning-facilitated attack [69] assessed useless user profile (Web search history)’s effectiveness in privacy protection. Kim et al. [70] first developed a stacked hourglass deep learning algorithm specific for landmark detection in images and then proposed a Web application for automatic cephalometric analysis. Based on remote computation of DNN classifiers of temporomandibular joint osteoarthritis, de Dumast et al. [71] proposed a Web system for biomedical data storage, integration, and computation. There are two studies using CNN to assist melanoma image classification [72, 73]. Specifically, in Ref. [72], a trained CNN aided clinicians in “skin lesion classification and provided a rationale for studies of such classifiers in real-life settings, wherein clinicians could integrate additional information such as patient age and medical history into their decisions (p. 1)”. In Ref. [73], ConvNet and bidirectional long short-term memory (LSTM) and two classification models (i.e., DocClass and SenClass) analyzed information from various websites with satisfactory accuracies.

#### Personalization and monitoring

AI technologies have shown popularity in realizing personalization in Web-based applications and through Web mining. For example, Wu et al. [74] developed “a dynamic machine learning closed loop consisting of smart wearable devices, human body data measurement devices, and Internet-based intelligent systems (p. 10)” for convenient and personalized use in the smart health system. Garcia-Rudolph et al. [75] adopted cutting-edge cluster validity indices to form hierarchical, partitional, and model-driven cluster strategies and applied principal component analysis and random forests for dimensionality reduction. Forster et al. [35] proposed a dietary feedback system for “delivering consistent, personalized dietary advice in a multicenter study and evaluated the impact of automating the advice system, in which decision trees linked data on the nutritional intake to feedback messages (p. 1)”. Colombet et al. [27] proposed and assessed a knowledge specification method based on decision algorithm and decision trees in a Web decision support system to allow estimating risks and accessing recommendations according to clinical profiles. Expert systems are also used for promoting personalization. In Ref. [76], expert systems and Web search technologies were integrated into personal health records domains to trigger and monitor intelligent personal health records.

In terms of monitoring, there are scholars focusing on AI for health monitoring and surveillance in Web-based applications. For example, to support event-based surveillance and understand factors that make an article relevant, Abbood et al. [77] “extracted expert labels from a public health unit that screens online resources every day to train various machine learning models and perform key information extraction as well as relevance scoring on epidemiological texts (p. 1)”. In a Web application integrated with machine learning algorithms designed to monitor pregnancy [78], users accessed “calculators of baby percentile, period tracker, pregnancy calendar, and baby vaccination schedule (p. 1)”. By combining information extraction based on rule-based systems and machine learning algorithms for classifying or identifying information on diseases, an application for automatically extracting disease information on the Web [79] produced “epidemiological information on diseases, locations, dates, hosts, and number of cases for outbreaks mentioned in the news and social media articles (p. 3)”.

In terms of monitoring via medical imaging, Hu et al. [80] monitored harmful algal blooms in aquatic ecosystems via Web applications for “real-time tracking of red tides caused by the toxic dinoflagellate Karenia brevis by employing an interface with three types of satellite-imagery and numerical data products combined from different sources (p. 1282)”.

#### Medical imaging, relationship mining, question answering, medical data storage and publishing, and facilitating dialogue and conversation

There are other types of medical- and healthcare-related tasks that have concerned AI scholars, including medical imaging, relationship mining, question answering, medical data storage and publishing, and facilitating dialog and conversation. In medical imaging, scholars focus on dermoscopic melanoma image classification [72] and organ segmentation in computed tomography [81]. For example, to minimize the time and effort required for technical (e.g., image annotation) and legal tasks (e.g., de-identification), Trägårdh et al. [81] used CNNs to segment organs in computed tomography to “extract standardized uptake values from the corresponding positron emission tomography images (p. 1)”. In relationship mining, Zhang et al. [82] mined putative disorder–gene–drug relations concerning Parkinson's diseases using a gene–disorder–drug semantic relationship mining algorithm that queried the relations among a variety of entities from varied data sources. With the use of ontologies and semantic Web, Traverso et al. [14] developed prediction algorithms for personalized therapy by proposing a scalable big data architecture “based on data standardization to transform clinical data into findable, accessible, interoperable and reusable data (p. 854)”.

Also, there are scholars focusing on medical data storage and publishing using CNNs. For example, Potočnik et al. [83] developed “the first publicly accessible USOVA3D database of annotated ultrasound volumes with ovarian follicles (p. 1)” and introduced automatic follicle-detection models with the basis of directional three-dimensional wavelet transform and CNNs.

Furthermore, recurrent HNN and back-propagation neural networks are mainly used for medical data storage and publishing through medical imaging in Web information management. Lakshmi et al. [39] proposed HNN-integrated image encryption technologies to deal with a variety of attacks via continuous learning and updating, in which the back-propagation neural network generated “image-specific keys that increased the resiliency against hackers and then the generated keys were used as an initial seed for confusion and diffusion sequence generation through HNN (p. 6671)”.

Additionally, NLP technologies are integrated into Web-based question answering systems to facilitate dialogue and conversation and speech recognition. For example, to facilitate question–answering in the medical domain, Abacha et al. [30] developed a semantic model with the basis of NLP for in-depth analysis of medical questions and documents. Amith et al. [84] proposed a software engine that harnesses patient health information dialog ontologies for dialog and contextual information management between agents and health consumers. In [85], a relative entropy-based sentence subset selection method promoted speech recognition error and language model perplexity.

#### Future research directions

Future research is encouraged focusing on algorithm and method innovations, additional information use, functionality improvement, practical use, model/system generalization, extension, evaluation, automation, and efficiency, data acquiring and quality improvement, allowing interaction, and facilitating collaboration. See Additional file 1: Table S3.

In terms of algorithm and method innovations, future efforts include: (1) using knowledge graphs to analyze medical information strongly relevant to expert knowledge to boost prediction performance [86], (2) adopting ontology-based methods to perform complex plan-oriented counseling and communication tasks [84], (3) utilizing CNNs and LSTM-CNN with diverse embedding and optimization technologies for epidemic outbreak analysis [44], (4) applying semi-automatic approaches to promote personalized healthcare information provided to facilitate users’ daily activities of living [76], (5) integrating additional security technologies like hashing to avoid malicious attackers [57], (6) using a combination of algorithms such as genetic algorithms and SVMs to facilitate accurate feature selection [55], and (7) adding more privacy-preserving statistics and machine learning algorithms to extensively promote flexibility in secure multicenters [57]. Additionally, there are scholars indicating the need to: (1) propose visual approaches to explore “the dyadic interaction between coaches and participants to better understand how to provide support and guidance to participants (p. 14) [61]”, and (2) analyze, mine, and extract Web page content by adopting machine learning algorithms and through quality information visualization within search engines [87].

In terms of additional information use, future studies can consider additional ontologies, features, new data sources and features, demographic information, additional types of mappings or services, and multiple context information. First, Falkman et al. [34] mentioned exploiting semantic Web-based foundation by using domain ontology and reasoning and by adding user and organizational ontologies; Tao et al. [88] highlighted the need to allow ontology import in the Web ontology language; and Traverso et al. [14] indicated using radiation oncology ontology “combined with other ontologies under development to combine and link DICOM information, clinical data and quantitative features computed on patients’ images and variables (p. 861)”. Second, Kim et al. [73] suggested identifying and using additional features or entities (e.g., diseases, places, and time) that are important for determining “whether a report mentions an infectious disease outbreak (p. 12)” in deep learning models; Peral et al. [89] indicated including new data sources like social networks; Chen et al. [50] highlighted adding new features to facilitate annotating nodules on computed tomography images; Motlagh et al. [53] mentioned adding features like NLP and the provision of consultancy services, psychotherapy, and medication. Third, Sahu et al. [90] mentioned collecting “demographic information from a subset of existing ecobee users to understand the association between age, sex, and other relevant demographic indicators (p. 8)”, and Arsevska et al. [79] indicated integrating geographical and language factors. Other directions include: (1) integrating additional physiological signal monitoring modules [91], (2) combining the temporality of messages in clustering [61], (3) adopting linked open data as complementary answer sources [30], (4) exploiting health data produced via passive smartphone sensing technologies and linking them with Web-based applications [92], (5) integrating additional types of mappings or services with the basis of clinical guidelines to allow linking electronic health records with guideline-oriented decision support applications [93], (6) integrating multiple context information based on deep learning [94], (7) allowing seamless integration of data from varied sources or repositories [54], and (8) collecting propagation-related information and time series information to enhance model performance [86].

In terms of functionality improvement, future efforts focus on classification, annotation, relationship mining, extraction, prediction, correlation, clustering, verification, harmonization, guideline provision, and parameter updating. First, Arsevska et al. [79] mentioned enhancing classification for location disambiguation by considering complicated features; Khan et al. [69] indicated exploring the unsteady behavior of classification algorithms; Abbood et al. [77] suggested prioritizing classification regarding crawler detection, and Hosseini et al. [55] highlighted the use of fuzzy classification to promote crawler detection. Second, there are scholars focusing on correlation analysis, including: (1) conducting correlation analysis for the individual facial action units to understand the decoupling of these individual features [47], and (2) demonstrating potential correlations between “a person’s descriptions about wartime experiences in their blogs with the ensuing symptoms or disorders via Focus groups and medical records analysis (p. 6) [13]”. Third, scholars are also encouraged to: (1) add functionality for real-time image annotation during meetings and make the transition to Internet-based telephone services [34], (2) adopt a health-related misinformation detection framework to English health misinformation detection [86], (3) explore repositioning drugs according to semantic relations for varied syndromes, for example, Parkinson’s diseases, Alzheimer’s diseases, and cancers [82], (4) facilitate the extraction of date and confirmed-case counts [77], (5) propose approaches for predicting user dropout rate to provide timely interventions accordingly [64], (6) extend system functionality by providing automatic graph-based summarization of input texts [56], (7) investigate “clustering solutions with a larger number of clusters or implementing additional features in the cluster analysis to represent other dimensions of participant experience (p. 14)” for richer characterization of participant experiences for personalization [61], (8) perform more intensive label harmonization using common data model ontologies [54], and (9) conduct syntactic analysis of natural language questions and test syntactic dependencies’ contribution on confirming previously extracted semantic relationships and detecting unfamiliar relationships [30]. Other directions include: (1) promoting “comprehensive care by establishing additional applications for home follow-ups and working with the children with the rare inherited disorders and their families (p. 11) [95]”, (2) developing neural-driven security solutions for multimedia data like color medical images, audios, and videos to be stored in the cloud [39], (3) updating parameters dynamically [96], and (4) improving predictors to reduce prediction bias to discover physiological mechanisms of ion channel-targeted conotoxins [41].

In terms of generalization and extension, future efforts should focus on general tool development by considering varied contexts and domains, increasing sample size, and exploiting alternative technologies. Methodologically speaking, researchers are encouraged to: (1) integrate service-oriented architecture systems to construct medical decision support systems that are “cross-platform, more comprehensive and of greater service value (p. 929) [97]”; and (2) explore diverse sensor technologies for model training and data collection [77]. To validate the generalizability of Web-based systems, future work should: (1) facilitate generalization of Web-driven question answering systems by considering “complex questions (e.g., why and when) and questions with new semantic relations not defined in reference ontologies (p. 592) [30]”, (2) integrate multilingual processing mechanisms to manage news sources in varied languages [73], (3) validate the effectiveness of systems on diverse diseases [98], (4) focus on multiple people and more motions [52], (5) explore varied consumer health domains such as medication adherence counseling, behavior changes, or mental health through additional dialogue ontology development [84], (6) apply to diverse contexts (e.g., regional hospitals and general specialist clinics) [99], (7) conduct model training on raw features with no moving window-based statistics [47], (8) test the approaches on varied modalities [47], (9) improve communication for applying federated learning in real-world medical data with multiple institutions [100], and (10) extend systems with ontologies relevant to users’ contextual information and their health behaviors to improve user experience [84]. There are also scholars focusing on the need to increase the sample size. For example, Bremer et al. [64] suggested replicating the results in a larger sample, Traverso et al. [14] indicated expanding the number of users and highlighted extending radiation oncology to ensure a fuller coverage, with: (1) detailed concepts to map radiation oncology annotations, and (2) detailed concepts to map treatment-associated concepts and properties like dose volume histograms, and Huang and Chen [33] pointed out ontology extension to integrate more topics (e.g., biomedical and western medicine). Additionally, in terms of general tool proposal, Falkman et al. [34] mentioned developing general tools to be applied in varied medical disciplines, and Ko et al. [101] indicated establishing real-time AI training systems to train models by adopting prospectively collected data worldwide.

In terms of model or system evaluation, future efforts need to focus on: (1) performing clinical trials to understand the impact of AI classifiers on skin cancer classification in real-life settings [72], (2) comparing with other predictive models via tenfold validation [98], (3) evaluating LSTM’s performance with Glove and Fasttext [44], (4) performing extrinsic evaluation focusing on the system’s ability “for high-risk findings in patient records and its impact on patient care and clinical decision-making (p. 318) [56]”, (5) validate summarization strategies with varied types of clinical texts (e.g., operative notes and radiology reports) in patient healthcare settings [56], (6) understanding how interaction patterns impact treatment benefits in Internet-based interventions [61], (7) exploring drug and side effect relationship extraction and adverse drug reaction extraction [62], (8) system assessment from different perspectives (e.g., group size and user profile size) [69], (9) studying the significance of adopting single variable versus multiple variables by search engines [87], and (10) conducting biological and clinical experiments to validate utility and effectives [54].

Additionally, other directions include: (1) practical use, for example, evaluating clinical decision support systems in real-world contexts to see how effective it supports clinical diagnosis and diminish diagnostic errors [31], adopting federated learning for actual medical data analysis through collaborations with multiple institutions [100], and designing Web-based tools for predicting the risk group of patients [67]; (2) automation, for example, automating the processes for improving data mining rules (e.g., rules acquired after implementing NLP technologies on Web data) [89], exploiting deep learning for automatic extraction of features from substantial log files [55], and automatic processing to infer health Web information quality to improve information retrieval [87]; (3) efficiency, for example, accelerating response time to enhance user experience [52], lengthening individual deployment duration to detect symptom occurrences with higher possibilities [102], and adopting graphics processing unit and field programmable gate array acceleration [57]; (4) data acquirement and quality improvement, for example, seizing physiological responses or symptoms for the exploration of the causal relationships between trigger exposure and asthma exacerbation [102], and constructing an inclusive database containing information on drug properties, dosage, and interactions related to depression medication [53]; (5) allowing interaction, for example, upgrading Web applications to allow users to input blood sample results along with the outcomes [101]; and (6) facilitating collaboration, for example, collaborating governmental healthcare sectors to construct text guidelines [27].

## Conclusion

AI algorithms, in conjunction with Web technologies, are promising to promote patient outcomes and facilitate healthcare processes. Literature reviews offer a clear summary of the available evidence concerning a particular research domain to recognize gaps and methodological concerns to inform improvements in its future development [103–105]. This study presents a first in-depth analysis of current advances in Web intelligence-driven human health literature. Such analysis is timely for the understanding of how healthcare is promoted with the help of Web intelligence and affords important insight into future directions. Results indicate an increasing interest in developing Web intelligence-based tools for human health. A large proportion of studies focus on system design and model development for disease detection and diagnosis, clinical/biomedical text mining, and prediction in Web-based applications, and Web mining and farming, where SVMs, CNNs, ANNs, random forests, decision trees, and semantic Web are the most popularly used algorithms. More specifically, we highlight the use of: (1) random forests, SVMs, and CNNs for disease detection and diagnosis, (2) semantic Web, ontology mining, and topic modeling for clinical or biomedical text mining, (3) ANNs and logistic regression for prediction, and (4) CNNs and SVMs for monitoring and classification. In addition, we also suggest future research on algorithm and method innovations, additional information use, functionality improvement, practical use, model/system generalization, extension, evaluation, automation, and efficiency, data acquirement, and quality improvement, allowing interaction, and facilitating collaboration. This study contributes to the field of Web intelligence by providing an in-depth understanding of Web intelligence-centered information technologies that can be applied to promote human health and smart healthcare. As brain informatics is brain sciences in the era of Web intelligence-centered information intelligence, where Web intelligence technologies play an important role in supporting brain science studies, findings of this study also contribute to the field of brain informatics by offering important insights into the effective and efficient applications of Web intelligence to support informatics-empowered brain studies.

## Supplementary Information

**Additional file 1: Table S1.** Search query. **Table S2.** Coding results. **Table S3.** Future directions on web intelligence-driven health research.

## Data Availability

The datasets used and/or analyzed during the current study are available from the corresponding author on reasonable request.

## Abbreviations

AI: Artificial intelligence
NLP: Natural language processing
CNNs: Convolutional neural networks
ANNs: Artificial neural networks
DNNs: Deep neural networks
LSTM: Long short-term memory
RNNs: Recurrent neural networks
AUC: Area under the curve
MSE: Mean-square error
SVMs: Support vector machines
AIDS: Acquired immune deficiency syndrome
HNN: Hopfield neural network
