# Supplementary material for: Machine and cognitive intelligence for human health: systematic review
Source: Brain Inform. 2022 Feb 12;9(1):5. doi: 10.1186/s40708-022-00153-9 (PMC8840949; doi:10.1186/s40708-022-00153-9)
Supplement: Supplementary file 1 — Additional file 1: Table S1. Search query. Table S2. Coding results. Table S3. Future directions on web intelligence-driven health research. [file 40708_2022_153_MOESM1_ESM.docx]

**Additional file 1**

**Table S1**. Search query

| Article type | Research article |
| --- | --- |
| Language | English |
| Databases | SCI-EXPANDED, SSCI |
| Time span | 2001-2020 |
| Web-related terms | "web"  OR “web-based” OR "website*"  OR "www"  OR "Internet" NOT "Internet of things" |
| Health-related terms | "medical"  OR "medicine"  OR "nursing"  OR "health*" OR "smart care" OR “[elder care](https://en.wikipedia.org/wiki/Internet_of_things#Elder_care)” |
| AI-related terms | "vision understanding" or "scene understanding" or "semantic*" or "k nearest*" or "fuzzy logic" or "neuro-fuzzy" or "text mining" or "deep network*" or "fuzzy system*" or "graph mining" or "neural fuzzy" or "neural-fuzzy" or "bayes network*" or "decision tree" or "deep learning" or "deep-learning" or "fuzzy control" or "kernel method" or "kinetic model" or "random forest" or "visual search" or "bayes learning" or "cloud robotics" or "face alignment" or "face detection" or "face synthesis" or "feature coding" or "fundus imaging" or "hybrid feature" or "naive bayesian" or "neural control" or "neural network*" or "robust control" or "computer vision" or "features mining" or "fuzzy reasoning" or "graph embedding*" or "hybrid coupling" or "knowledge graph*" or "learning kernel" or "regression tree*" or "retinal imaging" or "bayesian network*" or "face recognition" or "fuzzy clustering" or "fuzzy set theory" or "machine learning" or "machine-learning" or "naive bayes tree*" or "nearest neighbor*" or "nearest-neighbor*" or "pattern analysis" or "rough set theory" or "semantic mapping" or "genetic algorithm*" or "k-means*" or "language modeling" or "multiplex network*" or "nearest neighbor*" or "neural nets model*" or "ontology matching" or "pattern discovery" or "pattern selection" or "place recognition" or "scene recognition" or "sequence analysis" or "sequence learning" or "action recognition" or "bayesian inference" or "facial recognition" or "hesitant fuzzy set" or "human intelligence" or "image segmentation" or "intelligent system*" or "k-means clustering" or "k-nearest neigbour*" or "machine perception" or "object recognition" or "ontology alignment" or "question answering" or "sentiment analysis" or "speech recognition" or "swarm intelligence" or "affective computing" or "appearance modeling" or "automated reasoning" or "classification tree" or "deep belief network*" or "emotion recognition" or "fuzzy cognitive map*" or "image understanding" or "implicit cognition" or "intelligent control" or "machine translation" or "pattern recognition" or "ambient intelligence" or "brain-based learning" or "human-machine system*" or "image categorization" or "image classification" or "image reconstruction" or "image-based modeling" or "knowledge extraction" or "machine intelligence" or "multi-modal learning" or "scene classification" or "semantic orientation" or "tensor-train network*" or "information retrieval" or "intelligent computing" or "knowledge-based model*" or "man-machine interface*" or "visual classification" or "cross-view recognition" or "emotion classification" or "expression recognition" or "feature representation" or "gaussian mixture model*" or "information extraction" or "intuitive intelligence" or "pattern classification" or "reinforcement learning" or "support vector machine" or "support vector network*" or "artificial intelligence" or "association rule mining" or "bayesian belief network*" or "decision support system*" or "deep polynomial network*" or "ensemble classification" or "filter weights learning" or "intelligent computation" or "monte carlo tree search" or "proximal classification" or "representation learning" or "semantic topic analysis" or "semi-auto image tagging" or "support vectors machine" or "visual word recognition" or "association link network*" or "competitive intelligence" or "conditional random field*" or "evolutionary computation" or "fuzzy c-means clustering" or "image set classification" or "intelligent robot system*" or "multiple kernel learning" or "multiple-kernel-learning" or "named entity recognition" or "networked control system*" or "sentiment classification" or "bayesian learning" or "features learning" or "switched network cluster*" or "artificial neural-network*" or "causal structure learning" or "gradient boosting machine" or "pattern mining" or "word sense disambiguation" or "composite learning control" or "computational intelligence" or "deep contour-aware network*" or "feature detection" or "learning-based synchronous" or "multi-label classification" or "multiple-instance learning" or "naive bayes classification" or "neural dynamic programming" or "online sequential learning" or "fuzzy classification" or "gustafson-kessel clustering" or "multilingual knowledge base" or "natural language generation" or "natural language processing" or "content-based image retrieval" or "feature selection" or "support vector classification" or "intelligent learning" or "fuzzy knowledge representation" or "natural language understanding" or "artificial bee colony" or "artificial fish swarm algorithm*" or "artificial general intelligence" or "contrastive divergence learning" or "nearest subspace classification" or "nonnegative spectral clustering" or "spectral-spatial classification" or "imagery classification" or "novel intelligent damping controller" or "fuzzy inference" or "connectionist temporal classification" |

**Table S2**. Coding results

| Ref. | Publication year | Journal | WoS category | Countries/Regions | Institutions | Authors | Study design | Data | AI technologies | Clinical tasks | Performance evaluation | Scope of web intelligence |
| --- | --- | --- | --- | --- | --- | --- | --- | --- | --- | --- | --- | --- |
| [1] | 2020 | Journal of Ambient Intelligence and Humanized Computing | Computer Science, Artificial Intelligence; Computer Science, Information Systems; Telecommunications | China; Pakistan; UK | Xidian Univ; Univ Glasgow; COMSATS Inst Informat Technol | Shah, Syed Aziz; Fan, Dou; Ren, Aifeng; Zhao, Nan; Yang, Xiaodong; Tanoli, Shujaat Ali Khan | model development | Perturbations of the radio signal | SVM, random forests, k-nearest neighbors | disease detection and diagnosis | accuracy, precision, recall, F-score, statistical analysis | Web-based applications |
| [2] | 2020 | Information Sciences | Computer Science, Information Systems | China; Italy; Pakistan; South Korea | Kyungpook Natl Univ; Incheon Natl Univ; Xidian Univ; Univ Naples Federico II; Bahria Univ | Qureshi, Kashif Naseer; Din, Sadia; Jeon, Gwanggil; Piccialli, Francesco | model development | Clinical health records and physiological signals from wearable sensor nodes | ANN, SVM, naive bayes, decision trees, logistics regression, k-nearest neighbors | prediction | accuracy, sensitivity, specificity | Web-based applications |
| [3] | 2020 | EJNMMI Physics | Radiology, Nuclear Medicine & Medical Imaging | Sweden | Sahlgrens Univ Hosp; Univ Gothenburg; Chalmers Univ Technol; Eigenvision AB; RECOMI; Lund Univ; Skane Univ Hosp | Tragardh, Elin; Borrelli, Pablo; Kaboteh, Reza; Gillberg, Tony; Ulen, Johannes; Enqvist, Olof; Edenbrandt, Lars | system design | Annotated CT and PET/CT images | CNN | medical imaging | mean dice index | Web-based applications |
| [4] | 2020 | Expert Systems | Computer Science, Artificial Intelligence; Computer Science, Theory & Methods | Turkey | Firat Univ; Papatyasoft IT | Santur, Yunus; Santur, Sinem Guven; Karakose, Mehmet | system design | User data stored in a cloud-based application | ANN | monitoring | accuracy | Web-based applications |
| [5] | 2020 | BMC Medical Informatics and Decision Making | Medical Informatics | USA | Univ Texas Hlth Sci Ctr Houston; Kentucky Canc Registry; Univ Kent | Tao, Shiqiang; Zeng, Ningzhou; Hands, Isaac; Hurt-Mueller, Joseph; Durbin, Eric B.; Cui, Licong; Zhang, Guo-Qiang | system design | 47 out of 301 NAACCR data elements | ontology mapping | relationship mining | NA | ontological engineering |
| [6] | 2020 | BMC Bioinformatics | Biochemical Research Methods; Biotechnology & Applied Microbiology; Mathematical & Computational Biology | Italy | Natl Res Council; Univ Vita Salute San Raffaele; Inst Aerosp Med A Di Loreto | Stolfi, Paola; Valentini, Ilaria; Palumbo, Maria Concetta; Tieri, Paolo; Grignolio, Andrea; Castiglione, Filippo | model development | Time course of 46,170 virtual subjects | decision trees, random forests | prediction | MSE | Web-based applications |
| [7] | 2020 | BMC Medical Informatics and Decision Making | Medical Informatics | USA | Univ Texas Hlth Sci Ctr Houston; Washington Univ St Louis; Texas Technol Univ; Univ Texas Austin; S Methodist Univ | Amith, Muhammad; Lin, Rebecca Z.; Cui, Licong; Wang, Dennis; Zhu, Anna; Xiong, Grace; Xu, Hua; Roberts, Kirk; Tao, Cui | system design | dialogue script | ontology mapping | question answering | inter-rater agreement, accuracy | ontological engineering |
| [8] | 2020 | BMC Medical Informatics and Decision Making | Medical Informatics | China; USA | Tianjin Univ Sci & Technol; Univ Texas Hlth Sci Ctr Houston; Tianjin Key Lab Cognit Comp & Applicat; Tianjin Univ | Zhang, Li; Hu, Jiamei; Xu, Qianzhi; Li, Fang; Rao, Guozheng; Tao, Cui | model development | Life science datasets: SemMedDB, KEGG, Uniprot, and PharmGKB | semantic web | relationship mining | precision | Web mining and farming |
| [9] | 2020 | Journal of the European Academy of Dermatology and Venereology | Dermatology | Chile; Greece; South Korea; USA | Polytech Univ Catolica Chile; Dermatol Clin; Univ Athens; Mem Sloan Kettering Canc Ctr | Munoz-Lopez, C.; Ramirez-Cornejo, C.; Marchetti, M. A.; Han, S. S.; Del Barrio-Diaz, P.; Jaque, A.; Uribe, P.; Majerson, D.; Curi, M.; Del Puerto, C.; Reyes-Baraona, F.; Meza-Romero, R.; Parra-Cares, J.; Araneda-Ortega, P.; Guzman, M.; Millan-Apablaza, R.; Nunez-Mora, M.; Liopyris, K.; Vera-Kellet, C.; Navarrete-Dechent, C. | prospective diagnostic study | 380 skin conditions | DNN | disease detection and diagnosis | accuracy | Web-based applications |
| [10] | 2020 | IEEE Transactions on Consumer Electronics | Engineering, Electrical & Electronic; Telecommunications | China | Xidian Univ | Ding, Jianyang; Wang, Yong | system design | Human’s motions dataset in three different indoor environments with regard to laboratory, office room, and dormitory | RNN | disease detection and diagnosis | accuracy | Web-based applications |
| [11] | 2020 | Information Processing & Management | Computer Science, Information Systems; Information Science & Library Science | Germany; UK | Univ Augsburg; Imperial Coll London | Pandit, Vedhas; Schmitt, Maximilian; Cummins, Nicholas; Schuller, Bjorn | experimental design | The SEWA dataset | CNN | disease detection and diagnosis | MSE, mean absolute error, statistical analysis | Web mining and farming |
| [12] | 2020 | Scientific Programming | Computer Science, Software Engineering | Pakistan; Saudi Arabia | Univ Swabi; King Abdul Aziz Univ; Natl Univ Comp & Emerging Sci; Capital Univ Sci & Technol; Univ Agr | Khan, Rafiullah; Ahmad, Arshad; Alsayed, Alhuseen Omar; Binsawad, Muhammad; Islam, Muhammad Arshad; Ullah, Mohib | model development | Two subsets of an America Online dataset | J48, logistic model tree, decision Table, JRip, OneR, IBK, KStar, bagging, LogitBoost, Bayes Net, Rep tree | classification | precision | Web information retrieval |
| [13] | 2020 | Journal of Medical Internet Research | Health Care Sciences & Services; Medical Informatics | South Korea; USA | WonKwang Univ; Sungkyunkwan Univ; Mass Inst Technol; Wonkwang Univ Hosp; Chonnam Natl Univ; Univ Ulsan | Ko, Hoon; Chung, Heewon; Kang, Wu Seong; Park, Chul; Kim, Do Wan; Kim, Seong Eun; Chung, Chi Ryang; Ko, Ryoung Eun; Lee, Hooseok; Seo, Jae Ho; Choi, Tae-Young; Jaimes, Rafael; Kim, Kyung Won; Lee, Jinseok | model development | 361 COVID-19 patients in Wuhan, China, and applied it to 106 COVID-19 patients in three Korean medical institutions | DNN, random forests | prediction | sensitivity, specificity, accuracy, balanced accuracy | Web-based applications |
| [14] | 2020 | Nature Protocols | Biochemical Research Methods | USA | Michigan State Univ; Univ Calif San Francisco; Icahn Sch Med Mt Sinai; Univ Calif Davis | Zeng, Billy; Glicksberg, Benjamin S.; Newbury, Patrick; Chekalin, Evgeny; Xing, Jing; Liu, Ke; Wen, Anita; Chow, Caven; Chen, Bin | model development | 19,127 patient tissue samples covering more than 50 cancer types and expression profiles for 12,442 distinct compounds | autoencoder neural network | disease detection and diagnosis | statistical analysis | Web information management |
| [15] | 2020 | Frontiers in Public Health | Public, Environmental & Occupational Health | Switzerland | Univ Zurich | Grimm, Luisa A.; Bauer, Georg F.; Jenny, Gregor J. | randomized controlled trial | An initial sample of 22 teams in intervention group and 21 teams in control group from May 2018 to August 2019 | NA | facilitate dialogue and conversation | statistical analysis | Web-based applications |
| [16] | 2020 | BMC Medical Informatics and Decision Making | Medical Informatics | USA | Georgia Inst Technol; Emory Univ | Lee, Eva K.; Uppal, Karan | system design | CRAFT | random forests | clinical/biomedical text mining | accuracy | Web mining and farming |
| [17] | 2020 | Journal of Medical Internet Research | Health Care Sciences & Services; Medical Informatics | China | Zhejiang Univ | Lu, Yao; Zhou, Tianshu; Tian, Yu; Zhu, Shiqiang; Li, Jingsong | system design | 49,152 samples took approximately 7 minutes and 20 minutes | logistic regression | clinical/biomedical text mining | security analysis, accuracy loss, model training and evaluation time, scalability | Web-based applications |
| [18] | 2020 | PLOS ONE | Multidisciplinary Sciences | USA | Ctr Dis Control & Prevent; Georgia State Univ; SUNY Albany | Campo, David S.; Gussler, Joseph W.; Sue, Amanda; Skums, Pavel; Khudyakov, Yury | model development | Google Web-search volumes | extremely random forests | relationship mining | median absolute error | Web mining and farming |
| [19] | 2020 | Computational Biology and Chemistry | Biology; Computer Science, Interdisciplinary Applications | China | Inner Mongolia Univ | Sun, Zijie; Huang, Shenghui; Zheng, Lei; Liang, Pengfei; Yang, Wuritu; Zuo, Yongchun | experimental design | 673 RAACs generated from 74 types of reduced amino acid alphabet | SVM | disease detection and diagnosis | sensitivity, specificity, accuracy, statistical analysis | Web-based applications |
| [20] | 2020 | International Journal of Environmental Research and Public Health | Environmental Sciences; Public, Environmental & Occupational Health | South Korea | Catholic Univ Korea; Korea Inst Sci & Technol; Univ Sci & Technol | Kim, Mira; Chae, Kyunghee; Lee, Seungwoo; Jang, Hong-Jun; Kim, Sukil | model development | 2864 documents from various websites and subsequently manually categorized and labeled by two reviewers | CNN, RNN | classification | precision, recall, F-score, accuracy, AUC | Web mining and farming |
| [21] | 2020 | Computer Methods and Programs in Biomedicine | Computer Science, Interdisciplinary Applications; Computer Science, Theory & Methods; Engineering, Biomedical; Medical Informatics | Spain | Veratech Hlth SL; Univ Murcia; Univ Jaume 1 | Alberto Maldonado, Jose; Marcos, Mar; Tomas Fernandez-Breis, Jesualdo; Miguel Gimenez-Solano, Vicente; del Carmen Legaz-Garcia, Maria; Martinez-Salvador, Begona | system design | The first use case transforms clinical laboratory test results into an OWL representation in terms of a LOINC-based ontology, and the second one implements two colorectal cancer screening protocols | semantic web | clinical/biomedical text mining | NA | Web mining and farming |
| [22] | 2020 | PLOS Computational Biology | Biochemical Research Methods; Mathematical & Computational Biology | Czech Republic; Ireland; Spain | Univ Coll Dublin; Univ Navarra; Fujitsu Ireland Ltd; Natl Univ Ireland; Masaryk Univ | Novacek, Vit; McGauran, Gavin; Matallanas, David; Blanco, Adrian Vallejo; Conca, Piero; Munoz, Emir; Costabello, Luca; Kanakaraj, Kamalesh; Nawaz, Zeeshan; Walsh, Brian; Mohamed, Sameh K.; Vandenbussche, Pierre-Yves; Ryan, Colm; Kolch, Walter; Fey, Dirk | experimental design | Previously unknown phosphorylations by the LATS1, AKT1, PKA and MST2 kinases in human | knowledge graphs | prediction | sensitivity | Web-based applications |
| [23] | 2020 | BMC Health Services Research | Health Care Sciences & Services | USA | Icahn Sch Med Mt Sinai | Hu, Liangyuan; Li, Lihua; Ji, Jiayi; Sanderson, Mark | model development | The OCM data provided to Mount Sinai on 2938 breast-cancer episodes included both baseline periods and three performance periods between Jan 1, 2012 and Jan 1, 2018. | random forests | disease detection and diagnosis | accuracy, adaptability | Web mining and farming |
| [24] | 2020 | BMC Medical Genomics | Genetics & Heredity | UK | Univ Birmingham; Univ Hosp Birmingham; MRC Hlth Data Res UK HDR UK | Acharjee, Animesh; Larkman, Joseph; Xu, Yuanwei; Cardoso, Victor Roth; Gkoutos, Georgios V. | model development | Simulated data and publicly available datasets | random forests | disease detection and diagnosis | MSE | Web-based applications |
| [25] | 2020 | Neural Computing & Applications | Computer Science, Artificial Intelligence | India | SASTRA Deemed Univ | Lakshmi, C.; Thenmozhi, K.; Rayappan, John Bosco Balaguru; Rajagopalan, Sundararaman; Amirtharajan, Rengarajan; Chidambaram, Nithya | model development | Ciphered medical image database | recurrent hopfield neural network，back propagation neural network | medical data storage and publishing; medical imaging | statistical analysis | Web information management |
| [26] | 2020 | Frontiers in Genetics | Genetics & Heredity | South Korea | Seoul Natl Univ; Kyungpook Natl Univ; Chungbuk Natl Univ | Oh, Minsik; Park, Sungjoon; Lee, Sangseon; Lee, Dohoon; Lim, Sangsoo; Jeong, Dabin; Jo, Kyuri; Jung, Inuk; Kim, Sun | system design | Two time-series gene expression after drug treatment databases LINCS L-1000 and NCI-60 | ANN | clinical/biomedical text mining | sensitivity | Web-based applications |
| [27] | 2020 | Frontiers in Psychiatry | Psychiatry | Taiwan | Natl Yang Ming Univ; Taipei Vet Gen Hosp; Natl Def Med Ctr; Tao Yuan Psychiat Ctr | Chang, Yu-Wei; Tsai, Shih-Jen; Wu, Yung-Fu; Yang, Albert C. | system design | Two hundred schizophrenic patients and healthy controls in the Taiwan Aging and Mental Illness (TAMI) cohort | DNN | disease detection and diagnosis | accuracy, sensitivity, specificity | Web-based applications |
| [28] | 2020 | Journal of Medical Internet Research | Health Care Sciences & Services; Medical Informatics | France; Luxembourg | Luxembourg Inst Hlth; Paris Sud Paris Saclay Univ; Kap Code; Danone Nutricia Res | Schafer, Florent; Faviez, Carole; Voillot, Pamela; Foulquie, Pierre; Najm, Matthieu; Jeanne, Jean-Francois; Fagherazzi, Guy; Schuck, Stephane; Le Neve, Boris | retrospective infodemiology study | 36,989 different web users . A total of 29,935 messages (corresponding to 16,746 different web users) and 181,365 messages came from the Doctissimo subforum | topic modeling | clinical/biomedical text mining | expert evaluation | Web mining and farming |
| [29] | 2020 | PLOS Computational Biology | Biochemical Research Methods; Mathematical & Computational Biology | Germany | Robert Koch Inst; Inserve GmbH; Osnabruck Univ; Helmholtz Ctr Infect Res | Abbood, Auss; Ullrich, Alexander; Busche, Ruediger; Ghozzi, Stephane | model development | 61,320 epidemiological articles and the Wikipedia corpus | SVM, k-nearest neighbors, logistic regression, multilayer perceptron, CNN | monitoring | precision, sensitivity, specificity, F-score, index-balanced accuracy | Web-based applications |
| [30] | 2020 | Applied Sciences | Chemistry, Multidisciplinary; Engineering, Multidisciplinary; Materials Science, Multidisciplinary; Physics, Applied | China | Chinese Acad Sci; Hefei Normal Univ; Univ Sci & Technol China; Anhui Jianzhu Univ; Chuzhou Univ | Wu, Yichen; Ma, Zuchang; Zhao, Huanhuan; Li, Yibing; Sun, Yining | experimental design | 32 males and 29 females to undergo incremental cardiopulmonary exercise testing with cycling equipment | linear regression, random forests, elastic net, polynomial regression, ridge regression, and lasso regression | personalization | accuracy | Web mining and farming |
| [31] | 2020 | Computer Methods and Programs in Biomedicine | Computer Science, Interdisciplinary Applications; Computer Science, Theory & Methods; Engineering, Biomedical; Medical Informatics | Slovenia | LogicData; Univ Maribor; Univ Med Ctr Utrecht; IVF ADRIA Consulting | Potocnik, Bozidar; Munda, Jurij; Reljic, Milan; Rakic, Ksenija; Knez, Jure; Vlaisavljevic, Veljko; Sedej, Gasper; Cigale, Boris; Holobar, Ales; Zazula, Damjan | database development | USOVA3D testing dataset | CNN | medical data storage and publishing | inter-rater agreement | Web information management |
| [32] | 2020 | Journal of Medical Internet Research | Health Care Sciences & Services; Medical Informatics | Germany; USA | Leuphana Univ Luneburg; Univ Virginia | Bremer, Vincent; Chow, Philip, I; Funk, Burkhardt; Thorndike, Frances P.; Ritterband, Lee M. | NA | 151 participants from a fully automated web-based program (Sleep Healthy Using the Internet) | logistic regression, SVM, boosted decision trees | prediction | AUC | Web mining and farming |
| [33] | 2020 | Journal of Medical Internet Research | Health Care Sciences & Services; Medical Informatics | South Korea | Sungkyunkwan Univ; Samsung | Lee, Geun Hyeong; Shin, Soo-Yong | experimental design | Modified National Institute of Standards and Technology (MNIST), Medical Information Mart for Intensive Care-III (MIMIC-III), and electrocardiogram (ECG) datasets | federated learning | clinical/biomedical text mining | AUC, F-score, recall, precision | Web information management |
| [34] | 2020 | JMIR mHealth and uHealth | Health Care Sciences & Services; Medical Informatics | South Korea | Seoul Natl Univ; Keimyung Univ; Hansung Univ; Gyeonggi Univ Sci Technol | Kim, Jun-Min; Lee, Woo Ram; Kim, Jun-Ho; Seo, Jong-Mo; Im, Changkyun | system design | 2000 images randomly sampled from the images stored on the server | CNN | disease detection and diagnosis; medical imaging; monitoring | accuracy | Web-based applications |
| [35] | 2020 | Computational Intelligence and Neuroscience | Mathematical & Computational Biology; Neurosciences | China | Univ Sci & Technol Beijing; Harbin Inst Technol; Jiangxi Sci & Technol Normal Univ | Yan, Yongjie; Yu, Guang; Yan, Xiangbin | model development | 3856035 real votes, comments, and thank-you letters from 194.65 million patients in 605066 doctors’outpatient clinics in 9823 public hospitals across the country | CNN | clinical/biomedical text mining | precision, normalized discounted cumulative gain | Web mining and farming |
| [36] | 2020 | JMIR Medical Informatics | Medical Informatics | Spain | Fundacio Inst Invest Ciencies Salut Germans Trias; Inst Guttmann Hosp Neurorehabil; Univ Autonoma Barcelona | Garcia-Rudolph, Alejandro; Garcia-Molina, Alberto; Opisso, Eloy; Munoz, Jose Tormos | randomized controlled trial | 574 adult patients with TBI (mostly severe) undergoing web-based rehabilitation | principal component analysis, random forests | personalization | expert evaluation | Web mining and farming |
| [37] | 2020 | JMIR Medical Informatics | Medical Informatics | South Korea | Asan Med Ctr; Soongsil Univ; Univ Ulsan | Kim, Dong Wook; Kim, Kyung Won; Ko, Yousun; Park, Taeyong; Khang, Seungwoo; Jeong, Heeryeol; Koo, Kyoyeong; Lee, Jeongjin; Kim, Hong-Kyu; Ha, Jiyeon; Sung, Yu Sub; Shin, Youngbin | experimental design | 240 healthy participants (135 men and 105 women) with 40 participants per age group | CNN | disease detection and diagnosis | statistical analysis | Web-based applications |
| [38] | 2020 | Computer Methods and Programs in Biomedicine | Computer Science, Interdisciplinary Applications; Computer Science, Theory & Methods; Engineering, Biomedical; Medical Informatics | South Korea | Korea Inst Sci & Technol; Korea Univ Sci & Technol; Korea Univ Anam Hosp; Chungang Univ Hosp; Seoul Natl Univ; Yonsei Univ | Kim, Hannah; Shim, Eungjune; Park, Jungeun; Kim, Yoon-Ji; Lee, Uilyong; Kim, Youngjun | model development | 2,075 lateral cephalograms and ground truth positions of 23 landmarks from two institutes | CNN | classification | expert evaluation | Web-based applications |
| [39] | 2020 | Journal of Medical Internet Research | Health Care Sciences & Services; Medical Informatics | Germany | German Canc Res Ctr; Univ Hosp Cologne; Natl Ctr Tumor Dis; Univ Hosp Regensburg Univ Hosp Munich; Univ Kiel; Univ Erlangen Nurnberg; Univ Duisburg Essen; Univ Wurzburg; Univ Hosp Aachen; Heidelberg Univ; Berlin Inst Hlth; Charite | Maron, Roman C.; Utikal, Jochen S.; Hekler, Achim; Hauschild, Axel; Sattler, Elke; Sondermann, Wiebke; Haferkamp, Sebastian; Schilling, Bastian; Heppt, Markus, V; Jansen, Philipp; Reinholz, Markus; Franklin, Cindy; Schmitt, Laurenz; Hartmann, Daniela; Krieghoff-Henning, Eva; Schmitt, Max; Weichenthal, Michael; von Kalle, Christof; Frohling, Stefan; Brinker, Titus J. | experimental design | 100 unique dermoscopic images of melanomas and nevi | CNN | classification; medical imaging | sensitivity, accuracy | Web information management |
| [40] | 2020 | Applied Clinical Informatics | Medical Informatics | Canada; USA | Boston Univ; Brown Univ; Univ Toronto | Bala, Wasif; Steinkamp, Jackson; Feeney, Timothy; Gupta, Avneesh; Sharma, Abhinav; Kantrowitz, Jake; Cordella, Nicholas; Moses, James; Drake, Frederick Thurston | system design | Manually annotated corpus of 4,090 radiology reports from across our institution with a binary label indicating whether or not a report contains a newly discovered adrenal incidentaloma | CNN | disease detection and diagnosis | sensitivity, specificity, F-score | Web-based applications |
| [41] | 2020 | Decision Support Systems | Computer Science, Artificial Intelligence; Computer Science, Information Systems; Operations Research & Management Science | USA | Creighton Univ; Montclair State Univ; Univ S Dakota | Simsek, Serhat; Tiahrt, Thomas; Dag, Ali | model development | The records of 15,196 no-show patients and 57,406 show-up patients | ANN, genetic algorithm | classification | accuracy, sensitivity, specificity, AUC | Web-based applications |
| [42] | 2020 | IEEE Access | Computer Science, Information Systems; Engineering, Electrical & Electronic; Telecommunications | Pakistan; Saudi Arabia | Univ Jeddah; Kohat Univ Sci & Technol; King Abdul Aziz Univ | Amin, Samina; Uddin, M. Irfan; Zeb, M. Ali; Alarood, Ala Abdulsalam; Mahmoud, Marwan; Alkinani, Monagi H. | model development | dengue/ u corpus extracted from twitter by deploying Twitter Streaming API | Word2Vec, RNN | disease detection and diagnosis | accuracy, precision, recall, F-score, ROC | Web mining and farming |
| [43] | 2019 | Journal of Medical Systems | Health Care Sciences & Services; Medical Informatics | China | Sichuan Univ | Chen, Sihang; Guo, Jixiang; Wang, Chengdi; Xu, Xiuyuan; Yi, Zhang; Li, Weimin | system design | Two Pulmonary CT dataset, including DeepLN-I and DeepLN-II | DNN | disease detection and diagnosis | sensitivity, free-response receiver operating characteristics | Web-based applications |
| [44] | 2019 | Journal of Biomedical Semantics | Mathematical & Computational Biology | Spain; UK | Univ Mancheste; Hosp Salnes; Univ Liverpool; Univ Salford; Univ Polytech Madrid; Mid Cheshire Hosp NHS Fdn Trust; BMJ | Arguello-Casteleiro, Mercedes; Stevens, Robert; Des-Diz, Julio; Wroe, Chris; Fernandez-Prieto, Maria Jesus; Maroto, Nava; Maseda-Fernandez, Diego; Demetriou, George; Peters, Simon; Noble, Peter-John M.; Jones, Phil H.; Dukes-McEwan, Jo; Radford, Alan D.; Keane, John; Nenadic, Goran | model development | 11 well-known medical conditions mined from two sets of unstructured free-text data: 300 K PubMed Systematic Review articles (the PMSB dataset) and 2.5 M veterinary clinical notes (the VetCN dataset). | semantic web | clinical/biomedical text mining | F-score, precision, recall | Web mining and farming |
| [45] | 2019 | Applied Artificial Intelligence | Computer Science, Artificial Intelligence; Engineering, Electrical & Electronic | Algeria | Univ Blida1 | Fareh, Messaouda | model development | A written ontology of OWL, with incomplete knowledge | Bayesian network | disease detection and diagnosis | precision, recall, F-score | ontological engineering |
| [46] | 2019 | Journal of Medical Internet Research | Health Care Sciences & Services; Medical Informatics | USA | St Jude Childrens Res Hosp; Univ Washington; Seattle Childrens Res Inst | Chen, Annie T.; Swaminathan, Aarti; Kearns, William R.; Alberts, Nicole M.; Law, Emily F.; Palermo, Tonya M. | retrospective infodemiology study | Data from internet-delivered CBT arm (n=138) of the trial (N=273) | topic modeling | clinical/biomedical text mining | expert evaluation | Web mining and farming |
| [47] | 2019 | IEEE Access | Computer Science, Information Systems; Engineering, Electrical & Electronic; Telecommunications | China; UK | Beijing Univ Posts & Telecommun; Univ Sunderlan; Sichuan Univ | Liu, Yue; Yu, Ke; Wu, Xiaofei; Qing, Linbo; Peng, Yonghong | experimental design | 2,296 reliable and 2,085 unreliable health-related articles from multiple Chinese online social media sites | decision trees, SVM, k-nearest neighbors, AdaBoost, gradient boosting decision tree, random forests | disease detection and diagnosis | precision, F-score | Web mining and farming |
| [48] | 2019 | International Journal of Medical Informatics | Computer Science, Information Systems; Health Care Sciences & Services; Medical Informatics | Iran; Netherlands | Mashhad Univ Med Sci; Univ Amsterdam; Payame Noor Univ | Hosseini, Nafiseh; Fakhar, Fatemeh; Kiani, Behzad; Eslami, Saeid | experimental design | Real data of web sites selected from log files between 2010–2017 | SVM, bayesian network, decision trees | clinical/biomedical text mining | accuracy, F-score | Web information management |
| [49] | 2019 | International Journal of Medical Informatics | Computer Science, Information Systems; Health Care Sciences & Services; Medical Informatics | Brazil | W Parana State Univ; Univ Estadual Campinas | de Toledo, Thiago Ferreira; Lee, Huei Diana; Spolaor, Newton; Rodrigues Coy, Claudio Saddy; Wu, Feng Chung | system design | Audio files collected from 30 volunteers | ASR | clinical/biomedical text mining | accuracy | Web-based applications |
| [50] | 2018 | Medical Physics | Radiology, Nuclear Medicine & Medical Imaging | Netherlands | Maastricht Univ | Traverso, Alberto; van Soest, Johan; Wee, Leonard; Dekker, Andre | ontology development | The Radiation Oncology Ontology (ROO) contains 1,183 classes and 211 properties between classes to represent clinical data (and their relationships) in the radiation oncology domain following FAIR principles | semantic web | relationship mining | NA | ontological engineering |
| [51] | 2018 | PLOS ONE | Multidisciplinary Sciences | France; UK | French Natl Inst Agr Res; Univ Liverpool; Univ Montpellier | Arsevska, Elena; Valentin, Sarah; Rabatel, Julien; de Herve, Jocelyn de Goer; Falala, Sylvain; Lancelot, Renaud; Roche, Mathieu | system design | 352 disease-related news reports mentioning the diseases involved, locations, dates, hosts and the number of cases | SVM | monitoring | F-score, sensitivity | Web information management |
| [52] | 2018 | Disaster Medicine and Public Health Preparedness | Public, Environmental & Occupational Health | South Korea | Elect & Telecommun Res Inst; Seoul Natl Univ; Daumsoft; Samsung Fire & Marine Insurance | Woo, Hyekyung; Cho, Hyeon Sung; Shim, Eunyoung; Lee, Jong Koo; Lee, Kihwang; Song, Gilyoung; Cho, Youngtae | experimental design | Daily NAVER blog posts and Twitter posts from September 1, 2010 to June 30, 2014 using the social big-data-mining system, SOCIAL metricsTM (Daumsoft, Seoul, Korea). | SVM, random forests | disease detection and diagnosis | RMSE | Web mining and farming |
| [53] | 2018 | BMC Bioinformatics | Biochemical Research Methods; Biotechnology & Applied Microbiology; Mathematical & Computational Biology | India | Int Inst Informat Technol Hyderabad; Tata Consultancy Serv | Gupta, Shashank; Pawar, Sachin; Ramrakhiyani, Nitin; Palshikar, Girish Keshav; Varma, Vasudeva | model development | Twitter dataset annotated with ADR mention collected during the period of 2007-2010 | RNN | clinical/biomedical text mining | F-score, precision, recall | Web mining and farming |
| [54] | 2018 | Computerized Medical Imaging and Graphics | Engineering, Biomedical; Radiology, Nuclear Medicine & Medical Imaging | USA | Kitware Inc; Univ Michigan; Univ N Carolina Chapel Hill; Isomics Inc; Univ Texas MD Anderson Canc Ctr | de Dumast, Priscille; Mirabel, Clement; Cevidanes, Lucia; Ruellas, Antonio; Yatabe, Marilia; Ioshida, Marcos; Ribera, Nina Tubau; Michoud, Loic; Gomes, Liliane; Huang, Chao; Zhu, Hongtu; Muniz, Luciana; Shoukri, Brandon; Paniagua, Beatriz; Styner, Martin; Pieper, Steve; Budin, Francois; Vimort, Jean-Baptiste; Pascal, Laura; Prieto, Juan Carlos | system design | 259 condyles, 105 from control subjects and 154 from patients with diagnosis of TMJ OA | ANN | classification | expert evaluation | Web-based applications |
| [55] | 2018 | Clinical Otolaryngology | Otorhinolaryngology | UK | Wexham Pk Hosp; Gain Theory | Lau, K.; Wilkinson, J.; Moorthy, R. | system design | 1075 patients with 2-week head and neck cancer referrals from general practitioners | logistic regression | disease detection and diagnosis | precision, recall, F-score | Web-based applications |
| [56] | 2018 | IEEE Access | Computer Science, Information Systems; Engineering, Electrical & Electronic; Telecommunications | Spain | Univ Alicante | Peral, Jesus; Ferrandez, Antonio; Gil, David; Munoz-Terol, Rafael; Mora, Higinio | system design | Web data (the speci c context of two websites: PubMed and PLOS ONE), Structured database (the UCI database), Sensor data | ontology mapping, linear regression, SVM | personalization | accuracy | Web mining and farming |
| [57] | 2018 | PLOS ONE | Multidisciplinary Sciences | China | SE Univ | Nan, Yongqing; Gao, Yanyan | model development | Monthly time series data from January 2011 to June 2017 in the National Health and Family Planning Commission of China | ANN | prediction | median absolute error, RMSE, index of agreement | Web mining and farming |
| [58] | 2018 | Applied Intelligence | Computer Science, Artificial Intelligence | Iran | Iran Univ Sci & Technol; Kermanshah Univ Med Sci; Qom Univ | Motlagh, Hassan Ali Mohammadi; Bidgoli, Behrouz Minaei; Fard, Ali Akbar Parvizi | system design | 247 students and teachers from technical and vocational schools | fuzzy logic | disease detection and diagnosis | sensitivity, specificity | Web-based applications |
| [59] | 2018 | Online Information Review | Computer Science, Information Systems; Information Science & Library Science | Portugal | Univ Porto | Oroszlanyova, Melinda; Lopes, Carla Teixeira; Nunes, Sergio; Ribeiro, Cristina | model development | An annotated sample of 732 health web documents | logistic regression | prediction | accuracy | Web mining and farming |
| [60] | 2018 | Neurocomputing | Computer Science, Artificial Intelligence | Taiwan | Hwa Hsia Univ Technol | Chen, You-Shyang | model development | Two real datasets retrieved from the PSRS of existing hospital databases | decision trees | disease detection and diagnosis | AUC, ROC, specificity | Web-based applications |
| [61] | 2016 | PLOS ONE | Multidisciplinary Sciences | China; Singapore | Natl Univ Singapore; Zhejiang Chinese Med Univ; Chongqing Univ; Beijing Inst Technol | Li, Ying Hong; Xu, Jing Yu; Tao, Lin; Li, Xiao Feng; Li, Shuang; Zeng, Xian; Chen, Shang Ying; Zhang, Peng; Qin, Chu; Zhang, Cheng; Chen, Zhe; Zhu, Feng; Chen, Yu Zong | system design | Protein functional families | SVM, k-nearest neighbors, probabilistic neural network | prediction | sensitivity, specificity, precision, statistical analysis | Web-based applications |
| [62] | 2016 | Computer Methods and Programs in Biomedicine | Computer Science, Interdisciplinary Applications; Computer Science, Theory & Methods; Engineering, Biomedical; Medical Informatics | Taiwan | Taipei Med Univ; Natl Taipei Univ Nursing & Hlth Sci; Tainan Hosp | Rau, Hsiao-Hsien; Hsu, Chien-Yeh; Lin, Yu-An; Atique, Suleman; Fuad, Anis; Wei, Li-Ming; Hsu, Ming-Huei | experimental design | A sample (2060 cases) from the National Health Insurance Research Database of Taiwan | ANN | prediction | sensitivity, specificity, AUC | Web-based applications |
| [63] | 2016 | Journal of Medical Internet Research | Health Care Sciences & Services; Medical Informatics | Germany; Greece; Ireland; Netherlands; Norway; Poland; Spain; UK | Univ Navarra; Univ Coll Dublin; Newcastle Univ; Creme Global; Technol Univ Munich; Univ Oslo; Univ Reading; Natl Food & Nutr Inst; Harokopio Univ; Maastricht Univ | Forster, Hannah; Walsh, Marianne C.; O'Donovan, Clare B.; Woolhead, Clara; McGirr, Caroline; Daly, E. J.; O'Riordan, Richard; Celis-Morales, Carlos; Fallaize, Rosalind; Macready, Anna L.; Marsaux, Cyril F. M.; Navas-Carretero, Santiago; San-Cristobal, Rodrigo; Kolossa, Silvia; Hartwig, Kai; Mavrogianni, Christina; Tsirigoti, Lydia; Lambrinou, Christina P.; Godlewska, Magdalena; Surwillo, Agnieszka; Gjelstad, Ingrid Merethe Fange; Drevon, Christian A.; Manios, Yannis; Traczyk, Iwona; Alfredo Martinez, J.; Saris, Wim H. M.; Daniel, Hannelore; Lovegrove, Julie A.; Mathers, John C.; Gibney, Michael J.; Gibney, Eileen R.; Brennan, Lorraine | system design | 369 participants during the Food4Me randomized controlled trial | decision trees | personalization | expert evaluation | Web-based applications |
| [64] | 2016 | JMIR Medical Informatics | Medical Informatics | China; USA | HealthInfoNet; HBI Solut Inc; Stanford Univ; Pingjin Hosp Heart Ctr; Zhejiang Univ; Tsinghua Univ | Zheng, Le; Wang, Yue; Hao, Shiying; Shin, Andrew Y.; Jin, Bo; Ngo, Anh D.; Jackson-Browne, Medina S.; Feller, Daniel J.; Fu, Tianyun; Zhang, Karena; Zhou, Xin; Zhu, Chunqing; Dai, Dorothy; Yu, Yunxian; Zheng, Gang; Li, Yu-Ming; McElhinney, Doff B.; Culver, Devore S.; Alfreds, Shaun T.; Stearns, Frank; Sylvester, Karl G.; Widen, Eric; Ling, Xuefeng Bruce | model development | 1,385,280 notes representing 1,129,952 patients covering the period from July 1, 2012, to June 30, 2013, and 982,211 clinical notes representing 935,891 patients recorded from July 1, 2013, to June 30, 2014 | decision tree, ontology mapping | clinical/biomedical text mining | positive predictive value, sensitivity, specificity, negative predictive value, AUC | Web mining and farming |
| [65] | 2016 | IEEE Systems Journal | Computer Science, Information Systems; Engineering, Electrical & Electronic; Operations Research & Management Science; Telecommunications | USA | Florida Fish & Wildlife Conservat Commiss; Univ S Florida | Hu, Chuanmin; Murch, Brock; Corcoran, Alina A.; Zheng, Lianyuan; Barnes, Brian B.; Weisberg, Robert H.; Atwood, Karen; Lenes, Jason M. | model development | Three types of data products are fused: 1) near-real-time ocean color imagery tailored for red tide monitoring; 2) K. brevis cell abundance determined by sample analysis; and 3) ocean currents from a nested and validated numerical model | semantic web | monitoring; medical imaging | accuracy | Web-based applications |
| [66] | 2016 | In Vivo | Medicine, Research & Experimental | Greece; Italy; UK | Univ Aldo Moro; Univ Athens; Univ Oxford | Siristatidis, Charalampos; Vogiatzi, Paraskevi; Pouliakis, Abraham; Trivella, Marialenna; Papantoniou, Nikolaos; Bettocchi, Stefano | system design | Hospital data and IVF unit data | ANN | prediction | accuracy | Web-based applications |
| [67] | 2015 | Information Processing & Management | Computer Science, Information Systems; Information Science & Library Science | France; Luxembourg | Luxembourg Inst Sci & Technol; LIMSI CNRS | Ben Abacha, Asma; Zweigenbaum, Pierre | experimental design | MEDLINE 2001 and 8 semantic relations between diseases and treatments | semantic web | question answering | recall, precision, F-score | Web-based applications |
| [68] | 2013 | Journal of Medical Internet Research | Health Care Sciences & Services; Medical Informatics | Taiwan | Natl Taiwan Univ; Natl Taiwan Univ Hosp; Natl Chiao Tung Univ | Chen, Wei-Hsin; Hsieh, Sheau-Ling; Hsu, Kai-Ping; Chen, Han-Ping; Su, Xing-Yu; Tseng, Yi-Ju; Chien, Yin-Hsiu; Hwu, Wuh-Liang; Lai, Feipei | system design | 347,312 newborn dried blood samples collected at the Center between 2006 and 2011 | SVM | disease detection and diagnosis | sensitivity, specificity, accuracy | Web-based applications |
| [69] | 2012 | Journal of Medical Systems | Health Care Sciences & Services; Medical Informatics | USA | IBM | Luo, Gang | system design | Weight loss in COPD patients, Unintentional weight gain caused by psychotropic medication usage | expert system | personalization | NA | Web-based applications |
| [70] | 2010 | BMC Medical Informatics and Decision Making | Medical Informatics | USA | Ctr Dis Control & Prevent | Yu, Wei; Liu, Tiebin; Valdez, Rodolfo; Gwinn, Marta; Khoury, Muin J. | experimental design | 1999-2004 data set from the National Health and Nutrition Examination Survey | SVM | prediction | sensitivity, specificity, positive predictive value, negative predictive value | Web-based applications |
| [71] | 2010 | Journal of Medical Internet Research | Health Care Sciences & Services; Medical Informatics | USA | Yale Univ | Konovalov, Sergiy; Scotch, Matthew; Post, Lori; Brandt, Cynthia | model development | 90 military blog posts describing deployment situations and 60 control posts of Operation Enduring Freedom/Operation Iraqi Freedom (OEF/OIF) | bag-of-words, ontology mapping | clinical/biomedical text mining | precision, recall, F-score, accuracy, AUC | Web mining and farming |
| [72] | 2009 | Preventive Veterinary Medicine | Veterinary Sciences | Argentina; UK; USA | Univ Arizona; Inst Anim Hlth; Univ Calif Davis; Univ Natl Rosario | Perez, Andres M.; Zeng, Daniel; Tseng, Chun-ju; Chen, Hsinchun; Whedbee, Zachary; Paton, David; Thurmond, Mark C. | system design | FMD BioPortal from the World Reference Laboratory, including 8270 records from 122 countries between 1957 and 2007 | spatial scan statistic, risk-adjusted nearest neighbor hierarchical clustering, risk-adjusted vector clustering | disease detection and diagnosis | statistical analysis | Web-based applications |
| [73] | 2009 | IEEE/ACM Transactions on Audio, Speech, and Language Processing | Acoustics; Engineering, Electrical & Electronic | USA | Univ S Calif | Sethy, Abhinav; Georgiou, Panayiotis G.; Ramabhadran, Bhuvana; Narayanan, Shrikanth | experimental design | The English ASR of the Transonics English–Persian speech-to-speech translation system for doctor–patient interactions developed at USC. IBM’s speech recognition system for English, submitted to the 2006 evaluation within the TC-STAR project | ASR, n-gram | facilitate dialogue and conversation | perplexity | Web information retrieval |
| [74] | 2008 | Journal of General Internal Medicine | Health Care Sciences & Services; Medicine, General & Internal | USA | SUNY Stony Brook; Univ Calif Los Angeles | Grober, Mark L.; Mathew, Ashlei | system design | 50 consecutive Internal Medicine case records published in the New England Journal of Medicine | NA | disease detection and diagnosis | accuracy | Web-based applications |
| [75] | 2008 | Journal of Medical Internet Research | Health Care Sciences & Services; Medical Informatics | Sweden | Univ Skovde; Chalmers Univ Technol; Univ Gothenburg | Falkman, Goran; Gustafsson, Marie; Jontell, Mats; Torgersson, Olof | system design | 90 registered users located at 48 clinics | semantic web | clinical/biomedical text mining | NA | Web mining and farming |
| [76] | 2008 | Neural Network World | Computer Science, Artificial Intelligence | Turkey | TOBB Ekon Technol Univ | Ubeyli, Elif Derya | model development | Demographic and medical data of diabetics and non-diabetics obtained via the Internet | ANN | prediction | accuracy | Web-based applications |
| [77] | 2007 | Expert Systems with Applications | Computer Science, Artificial Intelligence; Engineering, Electrical & Electronic; Operations Research & Management Science | Taiwan | Natl Changhua Univ Educ | Huang, Mu-Jung; Chen, Mu-Yen | system design | The databases include a symptom database, a Chinese herbal medicine information database, a digestive system disease database, a disease treatment and prevention database, and image database. | ontology mapping, expert system | disease detection and diagnosis | expert evaluation | Web-based applications |
| [78] | 2007 | Bioinformatics | Biochemical Research Methods; Biotechnology & Applied Microbiology; Computer Science, Interdisciplinary Applications; Mathematical & Computational Biology; Statistics & Probability | USA | Univ Michigan | Xiang, Zuoshuang; Minter, Rebecca M.; Bi, Xiaoming; Woolf, Peter J.; He, Yongqun | system design | Synthetic data and laboratory research data | Bayesian network | prediction | accuracy | Web-based applications |
| [79] | 2007 | Diseases of the Colon & Rectum | Gastroenterology & Hepatology; Surgery | Italy; UK | Univ Dundee; Univ Perfezionamento; Univ Manchester | Dolgobrodov, S. G.; Moore, P.; Marshall, R.; Bittern, R.; Steele, R. J. C.; Cuschieri, A. | model development | A database (1,558 patients) by the Information & Statistics Division of National Health Service Scotland | ANN | prediction | ROC, statistical analysis | Web-based applications |
| [80] | 2005 | International Journal of Medical Informatics | Computer Science, Information Systems; Health Care Sciences & Services; Medical Informatics | France | Univ Paris 05 | Colombet, I; Aguirre-Junco, AR; Zunino, S; Jaulent, MC; Leneveut, L; Chatellier, G | system design | Guidelines published by the French National Agency for Evaluation in Health (ANAES) | decision trees | personalization | expert evaluation | Web-based applications |
| [81] | 2005 | International Journal of Medical Informatics | Computer Science, Information Systems; Health Care Sciences & Services; Medical Informatics | Norway | Univ Hosp N Norway; UiT | Bellika, JG; Hartvigsen, G | system design | 12 electronic discharge letters from the Department of Oncology, University Hospital of North Norway | ANN | disease detection and diagnosis | recall, precision | Web-based applications |

**Table S3**. Future directions on web intelligence-driven health research

| Algorithms and methods | A combination of methods, like genetic algorithms with a SVM be used to have accurate feature selection. | [48] |
| --- | --- | --- |
|  | Examine how to use knowledge graph to analyze medical information that are strongly related with expert knowledge to boost prediction performance. | [47] |
|  | Utilize the CNN and LSTM-CNN approaches with different embedding and optimization techniques to analyze epidemic outbreaks | [42] |
|  | Use ontology-based approaches to do more sophisticated plan-based counseling and communication tasks | [7] |
|  | Integrate additional security technologies such as hashing to prevent malicious attackers from tampering with the ciphertexts | [17] |
|  | Add more privacy-preserving statistics and machine learning methods to facilitate considerably enhance flexibility in secure multicenter | [17] |
|  | Develop visual methods to examine the dyadic interaction between coaches and participants to better understand how to provide support and guidance to participants during the course of an intervention | [46] |
|  | Use semi-automatic methods to facilitate the provision of personalized healthcare information to facilitate users' daily activities of living by 1) developing a protocol for medical professionals to follow in compiling triggers, and discovering patterns from already compiled triggers and use these patterns to help compile more triggers | [69] |
|  | Analyzing, mining, and extracting the content of web pages by using machine-learning methods, and visualization of the quality information within a search engine | [59] |
| Adding information | Integrate additional physiological signal monitoring modules | [82] |
|  | Exploit Semantic Web-based foundation by using the domain ontology and reasoning (e.g., to inform the browsing of cases) and by adding user and organizational ontologies | [75] |
|  | Combine the temporality of messages and other types of participant actions in cluster analysis | [46] |
|  | Use Linked Open Data as a complementary answer source | [67] |
|  | Identify and use additional features (entities), such as disease, person, place, and time, that which are essential elements to determine whether a report mentions an infectious disease outbreak, in deep learning models | [20] |
|  | Collect propagation-related information and time-series information to enhance model performance | [47] |
|  | Include new data sources such as social networks | [56] |
|  | Exploit health-related data generated through passive smartphone sensing methods and link them with web-based applications | [15] |
|  | Collect demographic information from a subset of existing ecobee users to understand the association between age, sex, and other relevant demographic indicators | [83] |
|  | Add features such as natural language processing and the ability to provide consultancy services, psychotherapy, and medication, to make the system more user-friendly and complete | [58] |
|  | Add new features to make system even better for annotating nodules on CT images | [43] |
|  | Integrate additional types of mappings or services based on clinical guidelines to enable the linking of EHR data with guideline-based decision-support tools under the same umbrella | [21] |
|  | Allow importing ontologies in the Web Ontology Language | [5] |
|  | Integrate geographical and language factors | [51] |
|  | Use the ROO combined with other ontologies under development to combine and link: DICOM information, clinical data and quantitative features computed on patients’ images and variables | [50] |
|  | Integrate multiple context information based on deep learning framework | [35] |
|  | Allow for more seamless integration of data from other sources and repositories, such as the GEO, the Sequence Read Archive, the EBI EGA and Treehouse | [14] |
| Functionality improvement | Add functionality for real-time annotation of images during meetings and make the transition to Internet-based telephone services | [75] |
|  | Enhance classification model for location disambiguation with more complex features | [51] |
|  | Perform a syntactic analysis of the NL question and test the contribution of syntactic dependencies on two aspects: (i) conﬁrmation of previously extracted semantic relations and (ii) detection of unknown relations: syntactic dependencies (Subject-Verb-Object) can replace triples (Entity1-Relation-Entity | [67] |
|  | Conduct correlation analysis for the individual FAUs, s to help shed light on how decoupled these individual features are | [11] |
|  | Apply HMD to English health misinformation detection | [47] |
|  | Mine repositioning drugs based on semantic relationships for more disorders, such as PD, Alzheimer’s Disease, cancer | [8] |
|  | Improve the performance of key date and confirmed-case count extractions | [29] |
|  | Explore the unsteady behavior of classiﬁcation algorithms | [12] |
|  | Prioritize classiﬁcation with regard to crawler detection | [29] |
|  | Use fuzzy classiﬁcation to improve crawler detection ability | [48] |
|  | Identify true positive presence in the house | [83] |
|  | Develop methods that predict the likelihood of user dropout over the duration of an intervention to enable researchers to devote resources to those at the highest risk of dropping out | [32] |
|  | Extend functionality of the system by providing automated graph- based summarization of the input text | [16] |
|  | Demonstrate possible correlations between a person's descriptions of his or her wartime experiences in their blog with the ensuing symptoms or disorders by using Focus groups and medical records analysis | [71] |
|  | Promote comprehensive care by establishing additional applications for home follow-ups and working with the children with the rare inherited disorders and their families | [68] |
|  | Examine clustering solutions with a larger number of clusters or employing additional features in the cluster analysis to represent other dimensions of participant experience for richer characterization of participant experiences for the purposes of tailoring and personalization | [46] |
|  | Implement a so-called ’portable veriﬁcation system’ that the users could download to their computers to allow test validation locally, signing the results digitally, and uploading them to our portal | [31] |
|  | Perform more intensive harmonization of labels using common data model ontologies | [14] |
|  | Develop neural-assisted security solutions for multimedia data such as color medical image, audio, and video storage in the cloud | [25] |
|  | Provide guidelines to users on an appropriate environment for acquiring images and improve the algorithms based on continuously increasing numbers of images as the number of users of the product increases | [34] |
|  | Update parameters or optimal features of our model over time | [52] |
|  | improve predictor to reduce prediction bias to the discovery of the physiological mechanism of ion channel-targeted conotoxins | [19] |
| Practical use | Evaluate CDSS in more natural environments for its potential to support clinical diagnosis and reduce the rate of diagnostic error in medicine | [74] |
|  | Use classification algorithms, data analysis, and semantic learning algorithms on actual data with the applications used | [4] |
|  | Use FL for actual medical data through collaborations with multiple institutions | [33] |
|  | Design web-based tool in a way that it not only predicts the risk group (of no show) of the patient but also provides the best appointment date on which the patients no show risk score is minimized | [41] |
|  | Suitability of the study results for generalized application to other cases | [60] |
| Generalization | Develop SOMWeb into a general tool that builds online CoPs for other medical disciplines is an interesting prospect | [75] |
|  | Facilitate generalization in terms of 1) complex questions (e.g. why, when), and 2) questions with new semantic relations that are not deﬁned in reference ontology | [67] |
|  | Incorporate a multilingual processing component to deal with important news sources in other languages | [20] |
|  | Establish a real-time AI training system that can continue to train our model using prospectively collected data from all over the world | [13] |
|  | Conduct the study with other diseases like Traumatic Brain Injury (TBI), which is another serious disease worldwide in terms of its impact on human life | [2] |
|  | Focus on multiple people, more motions, recognition accuracies | [10] |
|  | Integrate the SOA system with other services developed using different programming languages or platforms, such as a heterogeneous treatment decision support system, to form a medical decision support system that is cross-platform, more comprehensive and of greater service value | [84] |
|  | Explore other sensor technologies to train machine learning algorithms and generate data | [29] |
|  | Replicate the results of this study in a larger sample | [32] |
|  | Explore other consumer health domains like medication adherence counseling, behavioral health change, or mental health by simply constructing and importing new dialogue ontologies | [7] |
|  | Expand the number of users to continue proposing the ontology as underlying architecture for advance modeling applications such as distributed learning | [50] |
|  | Integrate cluster analysis as the initial phase of an ITA process to allow for a straightforward extension of a similar approach to other medical conditions, for example, patients who had a stroke | [36] |
|  | Retain and retest the experimental data set when applying the proposed model to different kinds of background settings, such as regional hospitals and general specialist clinics | [60] |
|  | Train models on the raw FAU features without any moving window-based statistics | [11] |
|  | Test the approach on across other modalities, and explore it’s usefulness as an adaptive, explainable feature fusion methodology | [11] |
| Extension | Integrate related medical diagnostic system by CMDS Java Expert System Shell and experiences;  Extend the ontology to integrate more topics including: biomedical, western medicine, etc.;  Transform a new physicians’ information system of CMDS into knowledge management concepts and framework | [77] |
|  | Expand the client–server version of FL and improve communication for the application of FL in real-world medical data with multiple institutions | [33] |
|  | Extend the software engine with ontologies that are related to user contextual information and health behavior change models that can link to the PHIDO to improve user experience with the conversational agent | [7] |
|  | Extend the ROO to guarantee a particular a broader coverage for an extensive use in the radiation oncology field, with 1) Detailed concepts for mapping radiation oncology annotations including organ at risks, nodals, and 2) Detailed concepts for mapping treatment-related concepts and properties such as Dose Volume histograms (DVH) | [50] |
|  | Expand USOVA3D database to at least 100 volumes, annotated by a minimum of two raters | [31] |
| Evaluation | Perform clinical trials to investigate how AI-based classifiers affect skin cancer classification in a real-life setting in which an improved classifier is incorporated in the diagnostic routine | [39] |
|  | Test the predictive model with other predictive models and apply a 10-Fold validation | [2] |
|  | Evaluate the performance of LSTM with Glove and Fasttext | [42] |
|  | Perform extrinsic evaluation of the system focusing on the ability of the system for high-risk findings in patient records and the impact on patient care and clinical decision-making and further validation of the summarization strategies using different types of clinical text such as operative notes and radiology reports in a patient care setting | [16] |
|  | Understand the potential impact of interaction patterns on treatment benefit from internet-delivered interventions | [46] |
|  | Explore drug and side-effect (adverse-effect) mention relation extraction along with ADR extraction and seek to validate if both can be formulated in a multi-task learncing setup | [53] |
|  | Assess the proposed attack from diﬀerent perspectives, such as the impact of group size, the number of queries in a session, user proﬁle size, and others | [12] |
|  | Study the importance of using a single variable vs a group of variables, potentially, by search engines | [59] |
|  | Conduct extensive biological and clinical validation experiments to verify utility and efﬁcacy | [14] |
|  | Validation and identification of the expert feature selection for application to other cases | [60] |
| Automation | Automate the process for improving DM rules, including rules obtained after applying NLP techniques on Web data (Web rules) | [56] |
|  | Use deep learning to extract features from massive log ﬁles automatically | [48] |
|  | Automated processes to infer the quality of health information on the web to improve information retrieval in the health domain | [59] |
| Efficiency | Speed up the total response time to further improve the user’s experience | [10] |
|  | Lengthen the duration of individual deployments to increase the likelihood of observing the occurrences of symptoms | [85] |
|  | Improve efficiency using graphics processing unit or field programmable gate array acceleration. | [17] |
| Data acquiring and quality improvement | Capture physiological responses and symptoms to effectively study the causal relationship between trigger exposure and asthma exacerbation, and the progression of disease severity | [85] |
|  | Construct a comprehensive database of information associated with depression medication to cover basic information on drug properties and important details on dosage, drug interactions, etc | [58] |
| Allow interaction | Upgrade the web application to allow a user to input his or her blood sample results along with the outcome | [13] |
| Facilitate collaboration | Collaborate with the French National Agency for Evaluation in Health (ANAES) to deﬁne such a framework and use it at the stage of development of the text guideline | [80] |

**Reference**

1. Shah, S.A., Fan, D., Ren, A., Zhao, N., Yang, X., Tanoli, S.A.K.: Seizure episodes detection via smart medical sensing system. J. Ambient Intell. Humaniz. Comput. 1–13 (2018).

2. Qureshi, K.N., Din, S., Jeon, G., Piccialli, F.: An accurate and dynamic predictive model for a smart M-Health system using machine learning. Inf. Sci. (Ny). 538, 486–502 (2020).

3. Trägårdh, E., Borrelli, P., Kaboteh, R., Gillberg, T., Ulén, J., Enqvist, O., Edenbrandt, L.: RECOMIA—a cloud-based platform for artificial intelligence research in nuclear medicine and radiology. EJNMMI Phys. 7, 1–12 (2020).

4. Santur, Y., Santur, S.G., Karaköse, M.: Architecture and implementation of a smart‐pregnancy monitoring system using web‐based application. Expert Syst. 37, e12379 (2020).

5. Tao, S., Zeng, N., Hands, I., Hurt-Mueller, J., Durbin, E.B., Cui, L., Zhang, G.-Q.: Web-based interactive mapping from data dictionaries to ontologies, with an application to cancer registry. BMC Med. Inform. Decis. Mak. 20, 1–9 (2020).

6. Stolfi, P., Valentini, I., Palumbo, M.C., Tieri, P., Grignolio, A., Castiglione, F.: Potential predictors of type-2 diabetes risk: machine learning, synthetic data and wearable health devices. BMC Bioinformatics. 21, 1–19 (2020).

7. Amith, M., Lin, R.Z., Cui, L., Wang, D., Zhu, A., Xiong, G., Xu, H., Roberts, K., Tao, C.: Conversational ontology operator: patient-centric vaccine dialogue management engine for spoken conversational agents. BMC Med. Inform. Decis. Mak. 20, 1–17 (2020).

8. Zhang, L., Hu, J., Xu, Q., Li, F., Rao, G., Tao, C.: A semantic relationship mining method among disorders, genes, and drugs from different biomedical datasets. BMC Med. Inform. Decis. Mak. 20, 1–11 (2020).

9. Muñoz‐López, C., Ramírez‐Cornejo, C., Marchetti, M.A., Han, S.S., Del Barrio‐Díaz, P., Jaque, A., Uribe, P., Majerson, D., Curi, M., Del Puerto, C.: Performance of a deep neural network in teledermatology: a single‐centre prospective diagnostic study. J. Eur. Acad. Dermatology Venereol. 35, 546–553 (2021).

10. Ding, J., Wang, Y.: A WiFi-based Smart Home Fall Detection System using Recurrent Neural Network. IEEE Trans. Consum. Electron. 66, 308–317 (2020).

11. Pandit, V., Schmitt, M., Cummins, N., Schuller, B.: I see it in your eyes: Training the shallowest-possible CNN to recognise emotions and pain from muted web-assisted in-the-wild video-chats in real-time. Inf. Process. Manag. 57, 102347 (2020).

12. Khan, R., Ahmad, A., Alsayed, A.O., Binsawad, M., Islam, M.A., Ullah, M.: QuPiD attack: machine learning-based privacy quantification mechanism for PIR protocols in health-related web search. Sci. Program. 2020, (2020).

13. Ko, H., Chung, H., Kang, W.S., Park, C., Kim, S.E., Chung, C.R., Ko, R.E., Lee, H., Seo, J.H., Choi, T.-Y.: An Artificial Intelligence Model to Predict the Mortality of COVID-19 Patients at Hospital Admission Time Using Routine Blood Samples: Development and Validation of an Ensemble Model. J. Med. Internet Res. 22, e25442 (2020).

14. Zeng, B., Glicksberg, B.S., Newbury, P., Chekalin, E., Xing, J., Liu, K., Wen, A., Chow, C., Chen, B.: OCTAD: an open workspace for virtually screening therapeutics targeting precise cancer patient groups using gene expression features. Nat. Protoc. 16, 728–753 (2021).

15. Grimm, L.A., Bauer, G.F., Jenny, G.J.: A Digital Tool to Build the Capacity of Leaders to Improve Working Conditions Related to Psychological Health and Well-Being in Teams: Intervention Approach, Prototype, and Evaluation Design of the Web-Application “wecoach.” Front. Public Heal. 8, (2020).

16. Lee, E.K., Uppal, K.: CERC: an interactive content extraction, recognition, and construction tool for clinical and biomedical text. BMC Med. Inform. Decis. Mak. 20, 1–14 (2020).

17. Lu, Y., Zhou, T., Tian, Y., Zhu, S., Li, J.: Web-Based Privacy-Preserving Multicenter Medical Data Analysis Tools Via Threshold Homomorphic Encryption: Design and Development Study. J. Med. Internet Res. 22, e22555 (2020).

18. Campo, D.S., Gussler, J.W., Sue, A., Skums, P., Khudyakov, Y.: Accurate spatiotemporal mapping of drug overdose deaths by machine learning of drug-related web-searches. PLoS One. 15, e0243622 (2020).

19. Sun, Z., Huang, S., Zheng, L., Liang, P., Yang, W., Zuo, Y.: ICTC-RAAC: An improved web predictor for identifying the types of ion channel-targeted conotoxins by using reduced amino acid cluster descriptors. Comput. Biol. Chem. 89, 107371 (2020).

20. Kim, M., Chae, K., Lee, S., Jang, H.-J., Kim, S.: Automated Classification of Online Sources for Infectious Disease Occurrences Using Machine-Learning-Based Natural Language Processing Approaches. Int. J. Environ. Res. Public Health. 17, 9467 (2020).

21. Maldonado, J.A., Marcos, M., Fernández-Breis, J.T., Giménez-Solano, V.M., del Carmen Legaz-García, M., Martínez-Salvador, B.: CLIN-IK-LINKS: A platform for the design and execution of clinical data transformation and reasoning workflows. Comput. Methods Programs Biomed. 197, 105616 (2020).

22. Nováček, V., McGauran, G., Matallanas, D., Vallejo Blanco, A., Conca, P., Muñoz, E., Costabello, L., Kanakaraj, K., Nawaz, Z., Walsh, B.: Accurate prediction of kinase-substrate networks using knowledge graphs. PLoS Comput. Biol. 16, e1007578 (2020).

23. Hu, L., Li, L., Ji, J., Sanderson, M.: Identifying and understanding determinants of high healthcare costs for breast cancer: a quantile regression machine learning approach. BMC Health Serv. Res. 20, 1–10 (2020).

24. Acharjee, A., Larkman, J., Xu, Y., Cardoso, V.R., Gkoutos, G. V: A random forest based biomarker discovery and power analysis framework for diagnostics research. BMC Med. Genomics. 13, 1–14 (2020).

25. Lakshmi, C., Thenmozhi, K., Rayappan, J.B.B., Rajagopalan, S., Amirtharajan, R., Chidambaram, N.: Neural-assisted image-dependent encryption scheme for medical image cloud storage. Neural Comput. Appl. 1–14 (2020).

26. Oh, M., Park, S., Lee, S., Lee, D., Lim, S., Jeong, D., Jo, K., Jung, I., Kim, S.: DRIM: A web-based system for investigating drug response at the molecular level by condition-specific multi-omics data integration. Front. Genet. 11, (2020).

27. Chang, Y.-W., Tsai, S.-J., Wu, Y.-F., Yang, A.C.: Development of an Al-Based Web Diagnostic System for Phenotyping Psychiatric Disorders. Front. Psychiatry. 11, (2020).

28. Schäfer, F., Faviez, C., Voillot, P., Foulquié, P., Najm, M., Jeanne, J.-F., Fagherazzi, G., Schück, S., Le Nevé, B.: Mapping and Modeling of Discussions Related to Gastrointestinal Discomfort in French-Speaking Online Forums: Results of a 15-Year Retrospective Infodemiology Study. J. Med. Internet Res. 22, e17247 (2020).

29. Abbood, A., Ullrich, A., Busche, R., Ghozzi, S.: EventEpi—A natural language processing framework for event-based surveillance. PLoS Comput. Biol. 16, e1008277 (2020).

30. Wu, Y., Ma, Z., Zhao, H., Li, Y., Sun, Y.: Achieve Personalized Exercise Intensity through an Intelligent System and Cycling Equipment: A Machine Learning Approach. Appl. Sci. 10, 7688 (2020).

31. Potočnik, B., Munda, J., Reljič, M., Rakić, K., Knez, J., Vlaisavljević, V., Sedej, G., Cigale, B., Holobar, A., Zazula, D.: Public database for validation of follicle detection algorithms on 3D ultrasound images of ovaries. Comput. Methods Programs Biomed. 196, 105621 (2020).

32. Bremer, V., Chow, P.I., Funk, B., Thorndike, F.P., Ritterband, L.M.: Developing a Process for the Analysis of User Journeys and the Prediction of Dropout in Digital Health Interventions: Machine Learning Approach. J. Med. Internet Res. 22, e17738 (2020).

33. Lee, G.H., Shin, S.-Y.: Federated Learning on Clinical Benchmark Data: Performance Assessment. J. Med. Internet Res. 22, e20891 (2020).

34. Kim, J.-M., Lee, W.R., Kim, J.-H., Seo, J.-M., Im, C.: Light-Induced Fluorescence-Based Device and Hybrid Mobile App for Oral Hygiene Management at Home: Development and Usability Study. JMIR mHealth uHealth. 8, e17881 (2020).

35. Yan, Y., Yu, G., Yan, X.: Online Doctor Recommendation with Convolutional Neural Network and Sparse Inputs. Comput. Intell. Neurosci. 2020, (2020).

36. Garcia-Rudolph, A., Garcia-Molina, A., Opisso, E., Muñoz, J.T.: Personalized Web-Based Cognitive Rehabilitation Treatments for Patients with Traumatic Brain Injury: Cluster Analysis. JMIR Med. Informatics. 8, e16077 (2020).

37. Kim, D.W., Kim, K.W., Ko, Y., Park, T., Khang, S., Jeong, H., Koo, K., Lee, J., Kim, H.-K., Ha, J.: Assessment of Myosteatosis on Computed Tomography by Automatic Generation of a Muscle Quality Map Using a Web-Based Toolkit: Feasibility Study. JMIR Med. Informatics. 8, e23049 (2020).

38. Kim, H., Shim, E., Park, J., Kim, Y.-J., Lee, U., Kim, Y.: Web-based fully automated cephalometric analysis by deep learning. Comput. Methods Programs Biomed. 194, 105513 (2020).

39. Maron, R.C., Utikal, J.S., Hekler, A., Hauschild, A., Sattler, E., Sondermann, W., Haferkamp, S., Schilling, B., Heppt, M.V., Jansen, P.: Artificial Intelligence and Its Effect on Dermatologists’ Accuracy in Dermoscopic Melanoma Image Classification: Web-Based Survey Study. J. Med. Internet Res. 22, e18091 (2020).

40. Bala, W., Steinkamp, J., Feeney, T., Gupta, A., Sharma, A., Kantrowitz, J., Cordella, N., Moses, J., Drake, F.T.: A Web Application for Adrenal Incidentaloma Identification, Tracking, and Management Using Machine Learning. Appl. Clin. Inform. 11, 606–616 (2020).

41. Simsek, S., Tiahrt, T., Dag, A.: Stratifying no-show patients into multiple risk groups via a holistic data analytics-based framework. Decis. Support Syst. 132, 113269 (2020).

42. Amin, S., Uddin, M.I., Zeb, M.A., Alarood, A.A., Mahmoud, M., Alkinani, M.H.: Detecting Dengue/Flu Infections Based on Tweets Using LSTM and Word Embedding. IEEE Access. 8, 189054–189068 (2020).

43. Chen, S., Guo, J., Wang, C., Xu, X., Yi, Z., Li, W.: DeepLNAnno: a Web-Based Lung Nodules Annotating System for CT Images. J. Med. Syst. (2019). https://doi.org/10.1007/s10916-019-1258-9.

44. Arguello-Casteleiro, M., Stevens, R., Des-Diz, J., Wroe, C., Fernandez-Prieto, M.J., Maroto, N., Maseda-Fernandez, D., Demetriou, G., Peters, S., Noble, P.-J.M.: Exploring semantic deep learning for building reliable and reusable one health knowledge from PubMed systematic reviews and veterinary clinical notes. J. Biomed. Semantics. 10, 1–28 (2019).

45. Fareh, M.: Modeling incomplete knowledge of semantic web using Bayesian networks. Appl. Artif. Intell. 33, 1022–1034 (2019).

46. Chen, A.T., Swaminathan, A., Kearns, W.R., Alberts, N.M., Law, E.F., Palermo, T.M.: Understanding user experience: Exploring participants’ messages with a web-based behavioral health intervention for adolescents with chronic pain. J. Med. Internet Res. 21, e11756 (2019).

47. Liu, Y., Yu, K., Wu, X., Qing, L., Peng, Y.: Analysis and detection of health-related misinformation on Chinese social media. IEEE Access. 7, 154480–154489 (2019).

48. Hosseini, N., Fakhar, F., Kiani, B., Eslami, S.: Enhancing the security of patients’ portals and websites by detecting malicious web crawlers using machine learning techniques. Int. J. Med. Inform. 132, 103976 (2019).

49. de Toledo, T.F., Lee, H.D., Spolaôr, N., Coy, C.S.R., Wu, F.C.: Web System Prototype based on speech recognition to construct medical reports in Brazilian Portuguese. Int. J. Med. Inform. 121, 39–52 (2019).

50. Traverso, A., Van Soest, J., Wee, L., Dekker, A.: The radiation oncology ontology (ROO): Publishing linked data in radiation oncology using semantic web and ontology techniques. Med. Phys. 45, e854–e862 (2018).

51. Arsevska, E., Valentin, S., Rabatel, J., De Goër de Hervé, J., Falala, S., Lancelot, R., Roche, M.: Web monitoring of emerging animal infectious diseases integrated in the French Animal Health Epidemic Intelligence System. PLoS One. 13, e0199960 (2018).

52. Woo, H., Cho, H.S., Shim, E., Lee, J.K., Lee, K., Song, G., Cho, Y.: Identification of keywords from Twitter and web blog posts to detect influenza epidemics in Korea. Disaster Med. Public Health Prep. 12, 352–359 (2018).

53. Gupta, S., Pawar, S., Ramrakhiyani, N., Palshikar, G.K., Varma, V.: Semi-supervised recurrent neural network for adverse drug reaction mention extraction. BMC Bioinformatics. 19, 1–7 (2018).

54. de Dumast, P., Mirabel, C., Cevidanes, L., Ruellas, A., Yatabe, M., Ioshida, M., Ribera, N.T., Michoud, L., Gomes, L., Huang, C.: A web-based system for neural network based classification in temporomandibular joint osteoarthritis. Comput. Med. Imaging Graph. 67, 45–54 (2018).

55. Lau, K., Wilkinson, J., Moorthy, R.: A web‐based prediction score for head and neck cancer referrals. Clin. Otolaryngol. 43, 1043–1049 (2018).

56. Peral, J., Ferrandez, A., Gil, D., Munoz-Terol, R., Mora, H.: An ontology-oriented architecture for dealing with heterogeneous data applied to telemedicine systems. IEEE Access. 6, 41118–41138 (2018).

57. Nan, Y., Gao, Y.: A machine learning method to monitor China’s AIDS epidemics with data from Baidu trends. PLoS One. 13, e0199697 (2018).

58. Motlagh, H.A.M., Bidgoli, B.M., Fard, A.A.P.: Design and implementation of a web-based fuzzy expert system for diagnosing depressive disorder. Appl. Intell. 48, 1302–1313 (2018).

59. Oroszlányová, M., Lopes, C.T., Nunes, S., Ribeiro, C.: Predicting the quality of health web documents using their characteristics. Online Inf. Rev. (2018).

60. Chen, Y.-S.: Identification of the human-oriented factors influencing AERC from the Web services. Neurocomputing. 279, 27–47 (2018).

61. Li, Y.H., Xu, J.Y., Tao, L., Li, X.F., Li, S., Zeng, X., Chen, S.Y., Zhang, P., Qin, C., Zhang, C.: SVM-Prot 2016: a web-server for machine learning prediction of protein functional families from sequence irrespective of similarity. PLoS One. 11, e0155290 (2016).

62. Rau, H.-H., Hsu, C.-Y., Lin, Y.-A., Atique, S., Fuad, A., Wei, L.-M., Hsu, M.-H.: Development of a web-based liver cancer prediction model for type II diabetes patients by using an artificial neural network. Comput. Methods Programs Biomed. 125, 58–65 (2016).

63. Forster, H., Walsh, M.C., O’Donovan, C.B., Woolhead, C., McGirr, C., Daly, E.J., O’Riordan, R., Celis-Morales, C., Fallaize, R., Macready, A.L.: A dietary feedback system for the delivery of consistent personalized dietary advice in the web-based multicenter Food4Me study. J. Med. Internet Res. 18, e150 (2016).

64. Zheng, L., Wang, Y., Hao, S., Shin, A.Y., Jin, B., Ngo, A.D., Jackson-Browne, M.S., Feller, D.J., Fu, T., Zhang, K.: Web-based real-time case finding for the population health Management of Patients with Diabetes Mellitus: a prospective validation of the natural language processing–based algorithm with statewide electronic medical records. JMIR Med. Informatics. 4, e6328 (2016).

65. Hu, C., Murch, B., Corcoran, A.A., Zheng, L., Barnes, B.B., Weisberg, R.H., Atwood, K., Lenes, J.M.: Developing a smart semantic web with linked data and models for near-real-time monitoring of red tides in the Eastern Gulf of Mexico. IEEE Syst. J. 10, 1282–1290 (2015).

66. Siristatidis, C., Vogiatzi, P., Pouliakis, A., Trivella, M., Papantoniou, N., Bettocchi, S.: Predicting IVF outcome: a proposed web-based system using artificial intelligence. In Vivo (Brooklyn). 30, 507–512 (2016).

67. Abacha, A. Ben, Zweigenbaum, P.: MEANS: A medical question-answering system combining NLP techniques and semantic Web technologies. Inf. Process. Manag. 51, 570–594 (2015).

68. Chen, W.-H., Hsieh, S.-L., Hsu, K.-P., Chen, H.-P., Su, X.-Y., Tseng, Y.-J., Chien, Y.-H., Hwu, W.-L., Lai, F.: Web-based newborn screening system for metabolic diseases: machine learning versus clinicians. J. Med. Internet Res. 15, e98 (2013).

69. Luo, G.: Triggers and monitoring in intelligent personal health record. J. Med. Syst. 36, 2993–3009 (2012).

70. Yu, W., Liu, T., Valdez, R., Gwinn, M., Khoury, M.J.: Application of support vector machine modeling for prediction of common diseases: the case of diabetes and pre-diabetes. BMC Med. Inform. Decis. Mak. 10, 1–7 (2010).

71. Konovalov, S., Scotch, M., Post, L., Brandt, C.: Biomedical informatics techniques for processing and analyzing web blogs of military service members. J. Med. Internet Res. 12, e45 (2010).

72. Perez, A.M., Zeng, D., Tseng, C., Chen, H., Whedbee, Z., Paton, D., Thurmond, M.C.: A web-based system for near real-time surveillance and space-time cluster analysis of foot-and-mouth disease and other animal diseases. Prev. Vet. Med. 91, 39–45 (2009).

73. Sethy, A., Georgiou, P.G., Ramabhadran, B., Narayanan, S.: An iterative relative entropy minimization-based data selection approach for n-gram model adaptation. IEEE Trans. Audio. Speech. Lang. Processing. 17, 13–23 (2009).

74. Graber, M.L., Mathew, A.: Performance of a web-based clinical diagnosis support system for internists. J. Gen. Intern. Med. 23, 37–40 (2008).

75. Falkman, G., Gustafsson, M., Jontell, M., Torgersson, O.: SOMWeb: a semantic web-based system for supporting collaboration of distributed medical communities of practice. J. Med. Internet Res. 10, e25 (2008).

76. Übeyli, E.D.: Use of the internet in medical decision-making: A model developed for diabetes prediction. Neural Netw. World. 18, 427 (2008).

77. Huang, M.-J., Chen, M.-Y.: Integrated design of the intelligent web-based Chinese Medical Diagnostic System (CMDS)–Systematic development for digestive health. Expert Syst. Appl. 32, 658–673 (2007).

78. Xiang, Z., Minter, R.M., Bi, X., Woolf, P.J., He, Y.: miniTUBA: medical inference by network integration of temporal data using Bayesian analysis. Bioinformatics. 23, 2423–2432 (2007).

79. Dolgobrodov, S.G., Moore, P., Marshall, R., Bittern, R., Steele, R.J.C., Cuschieri, A.: Artificial neural network: predicted vs. observed survival in patients with colonic cancer. Dis. colon rectum. 50, 184–191 (2007).

80. Colombet, I., Aguirre-Junco, A.-R., Zunino, S., Jaulent, M.-C., Leneveut, L., Chatellier, G.: Electronic implementation of guidelines in the EsPeR system: a knowledge specification method. Int. J. Med. Inform. 74, 597–604 (2005).

81. Bellika, J.G., Hartvigsen, G.: The oncological nurse assistant: A web-based intelligent oncological nurse advisor. Stud. Health Technol. Inform. (2004). https://doi.org/10.3233/978-1-60750-949-3-573.

82. Chen, C.-M.: Web-based remote human pulse monitoring system with intelligent data analysis for home health care. Expert Syst. Appl. 38, (2011).

83. Sahu, K.S., Oetomo, A., Morita, P.P.: Enabling Remote Patient Monitoring Through the Use of Smart Thermostat Data in Canada: Exploratory Study. JMIR mHealth uHealth. 8, e21016 (2020).

84. Chang, C.C., Lu, H.: A SOA‐based medical diagnosis decision support system using the Bayesian theorem and web service technology. J. Chinese Inst. Eng. 32, 923–930 (2009).

85. Dong, Q., Li, B., Downen, R.S., Tran, N., Chorvinsky, E., Pillai, D.K., Zaghloul, M.E., Li, Z.: A Cloud-Connected NO 2 and Ozone Sensor System for Personalized Pediatric Asthma Research and Management. IEEE Sens. J. 20, 15143–15153 (2020).
